# Cross-modal representations of first-hand and vicarious pain, disgust and fairness in insular and cingulate cortex

**DOI:** 10.1038/ncomms10904

**Published:** 2016-03-18

**Authors:** Corrado Corradi-Dell'Acqua, Anita Tusche, Patrik Vuilleumier, Tania Singer

**Affiliations:** 1 Swiss Centre for Affective Sciences, University of Geneva, Geneva CH-1211, Switzerland; 2 Laboratory for Neurology and Imaging of Cognition, Department of Neurosciences and Clinic of Neurology, University Medical Center, Geneva CH-1211, Switzerland; 3 Department of Psychology, FPSE, University of Geneva, Campus Biotech, 9, Chemin des Mines, Geneva CH-1211, Switzerland; 4 Max Planck Institute for Human Cognitive and Brain Sciences, Department of Social Neuroscience, Leipzig DE-04103, Germany; 5 Caltech Emotion and Social Cognition Laboratory, Division of the Humanities and Social Sciences, California Institute of Technology, Los Angeles, California 91125, USA

## Abstract

The anterior insula (AI) and mid-anterior cingulate cortex (mACC) have repeatedly been implicated in first-hand and vicarious experiences of pain, disgust and unfairness. However, it is debated whether these regions process different aversive events through a common modality-independent code, reflecting the shared unpleasantness of the experiences or through independent modality-specific representations. Using functional magnetic resonance imaging, we subjected 19 participants (and 19 confederates) to equally unpleasant painful and disgusting stimulations, as well as unfair monetary treatments. Multivoxel pattern analysis identified modality-independent activation maps in the left AI and mACC, pointing to common coding of affective unpleasantness, but also response patterns specific for the events' sensory properties and the person to whom it was addressed, particularly in the right AI. Our results provide evidence of both functional specialization and integration within AI and mACC, and support a comprehensive role of this network in processing aversive experiences for self and others.

## Abstract (web summary)

Anterior insula (AI) and medial anterior cingulate cortex (mACC) are activated by self and vicarious pain, disgust and fairness, yet the overlap of these representations are not known. Here the authors provide evidence for shared neural codes in the left AI and mACC and distinct codes in the right AI.

## Graphical abstract

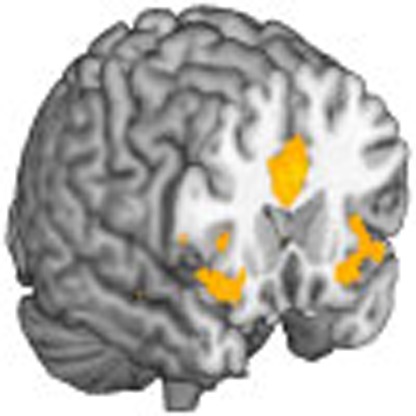

In the last decade, neuroscience research made considerable efforts to elucidate the neuronal processes underlying our ability to understand the mind of others^1^,^2^,^3^. Studies on the neural bases of empathy revealed that witnessing others' emotional states such as pain or disgust recruits a network comprising the anterior insula (AI) and mid-anterior cingulate cortex (mACC)^4^,^5^,^6^,^7^,^8^,^9^,^10^,^11^, both of which are also implicated in our first-person experience of the same aversive feelings^12^,^13^,^14^,^15^. Furthermore, witnessing transgressions of social norms (unfair transactions^16^ or unmoral acts^17^) has also been associated with activity in AI and mACC. These results represent an important cornerstone for embodied approaches of social cognition, according to which basal sensorimotor experiences, and their associated neural underpinnings, are instrumental in the processing of social events^18^,^19^,^20^.

It has been frequently proposed that neural signals in AI/mACC might not code for specific experiences (pain, disgust, unfairness, and so on), but instead mediate a broader function across different domains^9^,^10^,^21^. Unlike for the posterior and middle insula, that receive primary gustatory^22^,^23^ and nociceptive^24^,^25^,^26^ inputs from thalamic nuclei, the nature of the information coded by AI/mACC is still unclear. The sensitivity of these areas to different sensory and affective events^9^,^10^,^21^ might be interpreted in terms of a common neural coding of features such as unpleasantness, arousal or even the salience of the experience^27^,^28^. In particular, it has been suggested that pain-evoked activity in AI/mACC reflects a combination of neural processes similarly engaged by non-nociceptive events, arguing against the presence of any pain-specific^29^ (and possibly also disgust-specific) signal.

However, empirical evidence on the functional properties of AI/mACC has mainly been obtained by neuroimaging techniques such as functional magnetic resonance imaging (fMRI), whose spatial resolution does not allow recording from isolated neurons, but provides pooled signal from a large volume of grey matter. It is therefore possible that AI and mACC hold independent (but intermingled) neuronal populations that encode aversive states in a modality-specific (pain, disgust, unfairness) or even target-specific (self, other) manner.

In the present study, we investigated whether the neural processes subserved by AI/mACC are best described in terms of modality-specific representations of pain, disgust or unfairness, or rather in terms of modality-independent features shared by these different experiences, such as unpleasantness. To this end, we used multivoxel pattern analysis (MVPA) which allows for more detailed investigation of fMRI activation maps^30^,^31^,^32^. MVPA exploits the variability of neural signal over a cortical area that reflects the idiosyncratic distribution of a given neuronal population across neighbouring voxels. Thus, the identification of shared or independent activity patterns represents a more stringent assessment of whether two conditions recruit the same or different neuronal processes^30^,^31^,^32^,^33^. Using MVPA, we compared activity patterns from 19 female^7^ volunteers when they were subjected to either electrical (painful (Ps)/non-painful (nPs)) or gustatory stimuli (disgusting (Ds)/non-disguting (nDs)), which were carefully matched for subjective unpleasantness^34^ (self-related trials). In separate (other-related) trials, they saw a befriended confederate receiving equivalent electrical (Po/nPo) or gustatory (Do/nDo) stimuli^5^. Finally, participants also performed an Ultimatum Game (UG) task, in which they faced unknown people who proposed unfair or moderately fair economic transactions, either to the participants themselves (Us/Ms) or to the confederate (Uo/Mo)^16^,^35^,^36^,^37^,^38^,^39^.

First, we tested whether activity patterns evoked in AI/mACC by first-person painful experiences were similar to (or dissociated from) those evoked by first-person disgust, as a neural signature of modality-specific or modality-independent coding. Second, we assessed whether response patterns elicited by first-person experiences were shared with those elicited by vicarious pain/disgust, as well as unfairness. Third, and most importantly, we explored the nature of shared information for vicarious responses. If AI/mACC process others' affective states in terms of modality-specific features (‘it hurts when I see you in pain', ‘it is bitter when I see you disgusted'), then activity patterns evoked by a first-person event should generalize to vicarious experience of the same, but not different, modality. Alternatively, if AI/mACC process others' affective states in terms of modality-independent features (‘it is unpleasant when I see you in pain/disgusted'), then activity evoked by a first-person event should generalize to vicarious experiences of another modality. Finally, MVPA across different tasks could also shed light on the nature of the neural coding of unfairness in AI/mACC: if the role of these regions in unfairness reflects the engagement of disgust-specific neural responses (‘unfairness is disgusting'), as previously assumed^16^, then the activity patterns elicited during the UG should generalize to first-person disgust, but not pain. Alternatively, if AI/mACC code unfairness in terms of modality-independent features (‘unfairness is unpleasant'), then we expect shared information with both first-person pain and disgust.

Our results provide first evidence of both shared and independent coding in AI/mACC. In particular, we found that left AI and mACC disclose activity patterns which are shared between different modalities (pain, disgust and unfairness) but also between first-hand and vicarious aversive experiences, pointing to common coding of affective unpleasantness. Instead, right AI discloses activity patterns which are specific for the events' sensory properties and the target of the experience.

## Results

### Behavioural data

**Figure 1:**
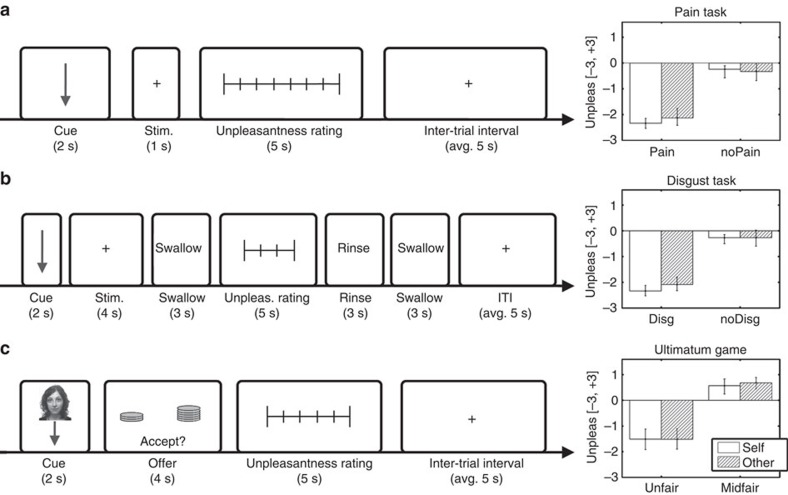
Trial structure and behavioural results. For (**a**) pain, (**b**) disgust and (**c**) ultimatum game (UG) tasks, trials were introduced by an arrow-shaped cue whose orientation and colour informed whether the upcoming event affected either the participants themselves (↓) or the confederate (↙). For the UG, cues were also associated with the photo of an unknown proposer. Cues (2 s) were followed by a stimulus event of varying unpleasantness (aversive versus neutral). For the pain task, this consisted of a 1-s-long electrical stimulation delivered to the left wrist. For the disgust task, 0.5 ml of liquid was delivered into the mouth, which had to be swallowed after 4 s when prompted by an instruction screen (3 s). To avoid transfer to the next trial, 2 ml of water were then delivered in the mouth (3 s), again followed by a 3-s swallowing instruction. For the UG, a 4-s screen was presented depicting the offer with 10 coins organized into 2 piles. At the bottom of the screen, the response options ‘accept/reject' were presented. For each task, stimuli were followed by a visual analogue scale, which participants used to rate the subjective unpleasantness evoked by the previous stimulation. Inter-trial intervals (ITI) in all three tasks varied from 2.5 to 7.5 s (average 5 s). For each task, average unpleasantness ratings are displayed in separate subplots (single experiment, sample size *N*=19). White bars refer to stimuli directed to the participants (self), whereas striped bars refer to stimuli directed to the confederates (other). For each subplot, aversive and neutral events are displayed separately along the horizontal axis. Error bars refer to bootstrap-based 95% confidence intervals.

Prior to scanning, electrical and gustatory stimuli were calibrated on an individual basis to ensure their painful or disgusting nature (see Methods). During the scanning session, participants rated the subjective unpleasantness associated with each stimulus event (see Fig. 1). The analysis of median unpleasantness ratings revealed that, for both the pain and disgust tasks, and for each target of the stimulation (self, other), aversive events were significantly more unpleasant than the corresponding neutral controls (paired *t*-tests on *N*=19, all *t*_(18)_s≥12.89 and *P*<0.001; see Fig. 1a,b). Furthermore, for both pain and disgust, the unpleasantness of aversive and neutral events was comparable across targets (paired *t*-tests all |*t*_(18)_s|≤1.92, not significant (NS), *N*=19). No modality-specific differences were found between pain and disgust, for each target and for each unpleasantness level (paired *t*-tests all |*t*_(18)_s|≤1.21, NS, *N*=19).

As the UG comprised a wide range of offers (most unfair, moderately fair, extremely fair), we used the unpleasantness ratings acquired during the scanning session to identify, on an individual basis, a sub-portion of UG trials that were most comparable with the pain and disgust tasks. Thus, for each participant, and for each target of the offer, we identified as ‘aversive' those UG trials that were rated as the most unpleasant, whereas the remaining trials whose unpleasantness was closest to 0 were identified as ‘neutral' (see Fig. 1c). Supplementary Figure 1 reports all details about the selected trials, with the most aversive being most frequently associated with unfair offers and neutral controls being associated with moderately fair (midfair) offers. As for pain and disgust, unpleasantness was matched across targets, both for unfair and midfair offers (paired *t*-tests all |*t*_(18)_s|≤1.46, NS, *N*=19). However, aversive and neutral events in the UG task were rated as slightly more positive than corresponding conditions in the pain and disgust tasks (paired *t*-tests all *t*_(18)_s≥2.35, *P*<0.05, *N*=19).

### Neural responses in AI and mACC

**Figure 2:**
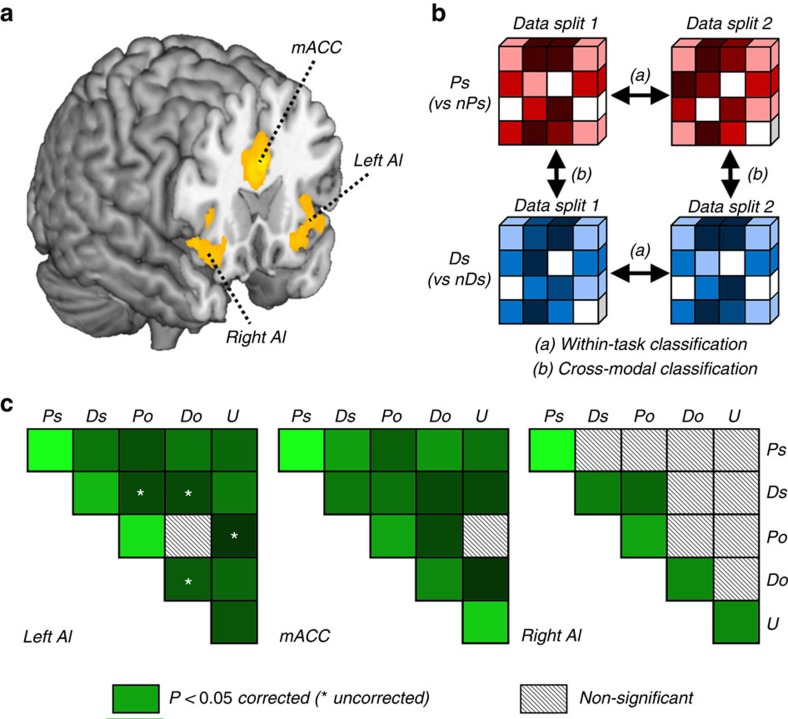
ROIs and MVPA overview. (**a**) Independent component analysis performed on participants' resting-state data, to map the AI–mACC network^28^ in an unbiased manner (surface rendering displayed at *P*<0.001). ROIs were based on significant clusters in the bilateral AI and mACC that were then used for MVPA on independent functional data obtained for the pain, disgust and UG tasks. (**b**) Processing steps in MVPA. For each ROI, data were extracted from every constitutive voxels and fed to a SVM classifier^40^,^62^, to identify multivoxel patterns informative about an aversive event relative to its corresponding neutral control (for example, Ps versus nPs). The so trained classifier was then tested on independent data from the same task (within-task classification) or another task, for example, when aversive experiences occur in a different sensory modality (cross-modal classification). (**c**) Matrixes displaying the results of within-task, cross-target and cross-modal classifications for each of the three ROIs. In each matrix, row and column labels refer to classification of an aversive state, each relative to its corresponding neutral control: self-related pain (Ps), self-related disgust (Ds), other-related pain (Po), other-related disgust (Do) and unfairness (U). *D'* values from within-task classifications are displayed in the outer diagonal line of the matrixes. The remaining cells in the matrixes reflect cross-target and cross-modal classifications. White striped cells refer to *d′* effects which are not significantly higher-than-chance, whereas green cells refer to effects significantly higher-than-chance. The luminance of green cells reflects the magnitude of the *d′* values. Single experiment, sample size *N*=19.

As first step, we focused on response patterns in AI and mACC, because these regions have repeatedly been implicated in both first-person and vicarious pain^5^,^7^,^8^,^12^,^13^, disgust^4^,^6^,^14^,^15^ and unfair UG offers^16^,^36^,^37^,^38^,^39^. Furthermore, these regions are part of an intrinsically connected circuit (called saliency network) that appears conserved even during rest^28^. We therefore took advantage of an independent resting-state session to localize this network in each subject and to obtain reliable regions-of-interest (ROIs) of AI and mACC without any bias related to tasks or stimuli. These ROIs provide subject-specific AI coordinates which are consistently connected with mACC and *vice versa*. See Fig. 2a for an illustration of the AI–mACC network at the group level, and Methods section for details.

As manipulation check, we investigated whether activation patterns in each ROI discriminated between aversive and non-aversive states (pain, disgust, unfairness), which could occur to participants themselves or the befriended confederate (within-task classification). A linear kernel support vector machine (SVM) classifier^40^ was trained on data from the respective task and condition, and then tested using cross-validation approaches. This led, for each participant, to a *d′* parameter that quantified the ability of the classifier to detect an aversive state as opposed to its neutral control (for example, Ps versus nPs). The significance of the *d′* values at the group level was estimated through rigorous permutation techniques (see Methods). In line with earlier studies using each task separately, we confirmed that the bilateral AI and mACC reliably encoded aversive states in all three modalities and both target-conditions. Repeated measure analysis of variances (ANOVAs) revealed no significant differences across ROIs in their sensitivity to self-/other-related pain and disgust. Regions differed, however, in their sensitivity to unfairness, reflecting greater sensitivity of mACC with respect to AI (see Table 1).

**Figure 3:**
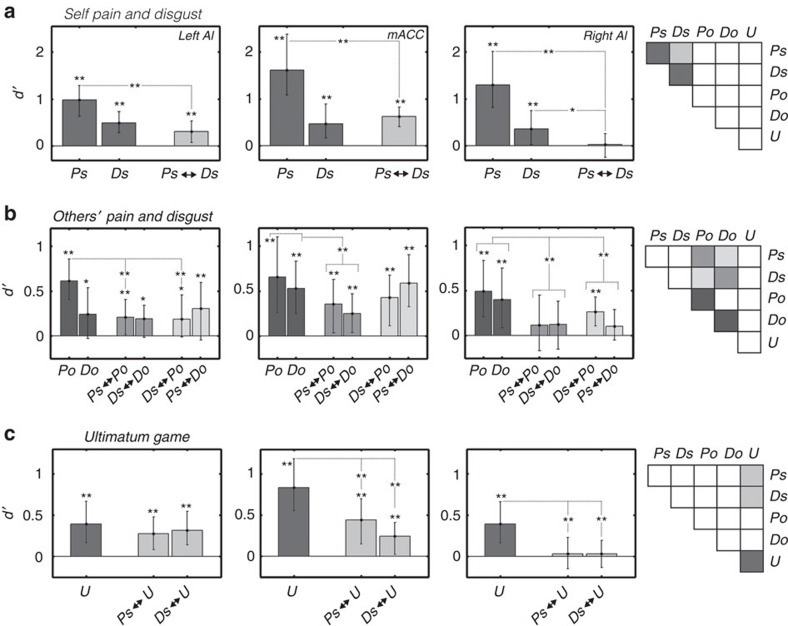
Bar plots displaying *d′* values from MVPA analyses in left AI, mACC and right AI. (**a**) Self-pain and -disgust. Bar plots display *d′* values representing the ability of a linear kernel SVM classifier to detect activity patterns characteristic for first-person experience of pain and disgust in ROIs in left AI, mACC and right AI. Ps and Ds refer to within-task classifications of self-related pain (Ps) and self-related disgust (Ds) compared with their respective neutral controls. Ps↔Ds refers to cross-modal classifications between both self-related aversive events. (**b**) Others' pain and disgust. *D'* values reflect within-task classification of other-related pain (Po) and disgust (Do) compared with their respective neutral controls, as well as cross-target classifications between aversive events directed at self and other for same modality (Ps↔Po; Ds↔Do) and different modality (Ds↔Po; Ps↔Do). (**c**) Ultimatum game. Bar plots display *d′* values related to the within-task classification of unfair events (U) compared with midfair events; and the cross-modal classification between unfairness and first-person pain (Ps↔U) or first-person disgust (Ds↔U). Bars are coloured consistently to the corresponding cells in the classification matrixes displayed in each panel. The significance of permutation tests comparing *d′* values against chance (or against values from other conditions) are also reported. **P<0.05 corrected for multiple comparisons for the three ROIs; *P<0.05 uncorrected. Error bars refer to bootstrap-based 95% confidence intervals. Single experiment, sample size *N*=19.

Subsequently, we ran a cross-modal pattern analyses to assess whether a classifier trained to detect one specific aversive event (for example, Ps versus nPs) could also detect aversiveness from an independent condition (for example, Ds versus nDs) and *vice versa* (see Fig. 2b for illustration). Reliable evidence for shared activity patterns between first-hand pain and disgust (Ps versus nPs↔Ds versus nDs; hereafter Ps↔Ds) was found in the left AI and mACC (*d′*s≥0.30; permutation-based test corrected for multiple comparisons for the 3 ROIs: cutoff=0.18, *P*<0.01, *N*=19, see Fig. 3a and Table 2). In contrast, no cross-modal effects were observed in the right AI (*d′*=0.02, NS), although this region reliably encoded both pain and disgust in the within-task classifications. Indeed, *d′s* in the right AI obtained for within-task classifications of both first-hand pain and disgust were significantly larger than those obtained for the cross-modal effect (Ps>PS↔Ds and Ds>Ps↔Ds; d′ differences (diff-*d′*s)≥0.33, permutation tests on *d′* differences: cutoffs≤0.29, *P*<0.05, *N*=19), thus suggesting the presence of modality-independent (but overlapping) activity patterns for pain and disgust. Results of a repeated measure ANOVA confirmed differences in cross-modal effect between first-hand pain and disgust across ROIs (permutation-based ANOVA: F=6.77, cutoff=3.40, *P*<0.01, *N*=19), reflecting lower *d′*s in right AI relative to both left AI and mACC (|diff-*d′*s|≥0.28, permutation tests on *d′* differences: |cutoffs|≤0.25, *P*<0.05, *N*=19). Overall, our data hint that the neural signal in left AI and mACC may be partly shared between pain and disgust, whereas the signal in right AI may instead reflect modality-specific coding.

Having established that AI/mACC disclose both modality-specific and modality-independent activity patterns for first-hand pain and disgust, we tested whether these neural responses were re-activated when the same experiences occurred to the confederate. This was achieved by assessing whether a classifier trained at detecting first-person pain (or disgust) could successfully detect others' pain (or disgust) and *vice versa* (cross-target classification: Ps↔Po, Ds↔Do). For left AI and mACC, significant effects were found for both pain and disgust (*d′*s≥0.21, permutation tests: cutoffs≥0.20, *P*<0.05 corrected for the 3 ROIs; except for Ds↔Do in left AI: *d′*=0.19, significant under an uncorrected cutoff=0.15, *P*<0.05, *N*=19). Critically, evidence in left AI and mACC for shared activity between first-hand and vicarious aversive experiences extended to stimuli of different modalities: that is, between first-person pain and vicarious disgust or first-person disgust and vicarious pain (Ds↔Po, Ps↔Do: *d′*s≥0.30, permutation tests: cutoffs≤0.20, *P*<0.01 corrected; except for Ds↔Po in left AI: *d′*=0.18, uncorrected cutoff=0.15, *P*<0.05, *N*=19). These findings reveal that vicarious pain and disgust elicit neural responses in left AI and mACC that are partly shared with those associated with first-hand experiences, regardless of whether the experience is of the same or different modality (see Fig. 3b).

Interestingly, we found no conclusive evidence of shared activity between first-hand and vicarious pain and disgust in right AI (*d′s*≤0.12 permutation tests: cutoffs≥0.19, NS; but for Ds↔Po: *d′*=0.26, *P*<0.05, *N*=19). However, the right AI disclosed reliable detection of pain and disgust in the within-task classification for both self and other (see Fig. 3a,b). Indeed, *d′*s obtained in right AI for the within-task classification of others' pain and disgust were greater than for the cross-target classifications between first-hand and vicarious experiences, both when the modality was the same (diff-*d′*=0.33, permutation test of *d′* differences: cutoff=0.25, *P*<0.01 corrected, *N*=19) or different (diff-*d′*=0.26, cutoff=0.25, *P*<0.05 corrected, *N*=19; see Fig. 3b). Taken together, these findings suggest a dissociation in neural responses between first-person and vicarious sensations for both pain and disgust in right AI.

Finally, we investigated whether a classifier trained to detect unfair (as opposed to marginally fair) UG offers could also detect first-person experiences of pain/disgust and *vice versa* (Ps↔Us, Ds↔Us, Ps↔Uo, Ds↔Uo). See Supplementary Fig. 2 for exhaustive representation of the results, which were comparable regardless of the target of the unfairness. For readability purposes, here we report effects identified when combining together self- and other-related UG trials, which indicated significant cross-modal effects of UG responses for both first-person pain and disgust in left AI and mACC (Ps↔U: *d′*s≥0.27, permutation test: cutoff=0.17; Ds↔U: *d′*s≥0.25, cutoff=0.16; *P*<0.05 corrected, *N*=19; see Fig. 3c). Moreover, *d′*s were comparable for cross-modal effects of UG patterns when compared with either pain or disgust (Ps↔U versus Ds↔U; |diff-*d′*s|≤0.20, permutation tests of *d′* differences: |cutoffs|≥0.23, NS, *N*=19), suggesting that neural representations of unfairness were not specifically related to disgust experiences. By contrast, no cross-modal effect was found in right AI (*d′*s≤0.04, NS), although this region disclosed reliable detection of unfairness in the within-task classification. Furthermore, in right AI, the *d′*s obtained in the within-task classification of unfairness were larger than those obtained in the cross-modal classification (U>Ps↔U and U>Ps↔U: diff-*d′*≥0.34, permutation tests of *d′* differences: cutoffs≤0.27, *P*<0.05 corrected, *N*=19). Finally, results of a repeated measure ANOVA confirmed differential cross-modal effects across ROIs for unfair UG offers and both first-hand pain (permutation-based ANOVA, F=4.56, cutoff=3.53, *P*<0.05, *N*=19) and disgust (permutation-based ANOVA, F=2.84, cutoff=3.16, *p* (one-tailed)<0.05, *N*=19), reflecting lower *d′*s in right AI than in both left AI and mACC (|diff-*d′*s|≥0.21, permutation tests of *d′* differences: |cutoffs|≤0.21, *P*<0.05, *N*=19).

Overall, the analysis of UG data points to shared activity patterns between pain, disgust and unfairness in left AI and mACC. These responses in the UG might reflect a general aversive affective experience shared with other primary negative experiences such as pain and disgust, rather than the activation of neuronal responses specific for a particular nociceptive or distasteful sensation. On the other hand, evidence from right AI suggests that part of the signal is specific to the experience of unfairness, which converges with our previous analyses for pain and disgust.

To summarize results from these analyses, we depicted for each ROI separately the *d′*-matrixes obtained from all within-task, cross-modal and cross-target classifications (Fig. 2c). A clear difference between the three ROIs is apparent, with the right AI displaying higher-than-chance *d′*s prevalently in within-task classifications (on the outer diagonal line), whereas the left AI and mACC displayed reliable *d′*s also in many cross-modal and cross-target classifications. Importantly, the absence of shared effects in right AI cannot be imputed to reduced signal or larger noise, as this region sensitivity to aversiveness in the within-task classification was comparable to that of other ROIs (in particular left AI; see also previous sections above).

### Whole-brain searchlight analysis

To test for additional brain regions relevant for the encoding of unpleasantness across targets and tasks, we repeated the pattern analyses using a whole-brain searchlight approach as implemented in other studies^7^,^32^,^33^,^41^,^42^. This approach has the advantage of not relying on *a priori* assumptions about specific brain regions but looks for discriminative local information throughout the whole brain. Results from this analysis were entirely in line with those obtained using ROIs. First of all, they confirmed the role of left AI and ACC in supramodal representations of negative affect, with shared activity patterns across all conditions. In addition, modality-independent effects were also observed in the posterior portion of the superior temporal sulcus and the inferior frontal gyrus, whereas the posterior cingulate cortex, supplementary motor area and orbitofrontal cortex exhibited modality-specific activity patterns. Please see Supplementary Figs 3–7 and Supplementary Tables 1–7 for more details.

## Discussion

Neuroscientific research has repeatedly shown that AI and mACC are engaged in first-person and vicarious experiences of pain^5^,^7^,^8^,^12^,^13^ and disgust^4^,^6^,^14^,^15^, but also unfairness^16^,^36^,^37^,^38^,^39^. However, the exact nature of the processes encoded in these regions is still debated^7^,^29^,^43^. Here we tested whether AI/mACC encode aversive states in terms of general affective features that are blind to the modality (pain, disgust, unfairness) and the target of the experience (self, other), or in terms of distinct modality-specific or target-specific neural responses. By using MVPA, we systematically compared the activity patterns elicited by painful (electrical), disgusting (gustatory) and unfair (monetary) events, directed at either the participant or another person. Notably, the left AI and mACC disclosed shared neural representations not only between first-person and vicarious experiences (extending previous MVPA results for pain^7^ to other domains), but also between different stimulus modalities including pain, disgust and unfairness. Furthermore, we also found neural responses specific to the modality and the target of the aversive experience, particularly in right AI. These results provide evidence for both domain-general and domain-specific coding in AI and mACC, thus demonstrating how this network might contribute to a comprehensive representation of aversive experiences.

Studies in primates and humans previously suggested that the insula might process first-hand experiences of pain and disgust in a sensory-specific manner. The posterior and middle insula are thought to receive inputs from neurons in ventral posteromedial thalamic nuclei sensitive to gustation^22^,^23^ and nociception^24^,^25^,^26^, and then to project to the AI for higher level coding of affect and interoceptive information^19^,^44^,^45^. However, the nature of the information coded in AI has still remained elusive. On the one hand, its sensitivity to a wide range of sensory and affective experiences^9^,^10^,^21^ suggests that AI (and mACC) may not code for modality-specific information, but rather represent more general properties of affective experiences common to pain, disgust and unfairness. On the other hand, anatomical studies on primates point to several distinct subfields in AI^46^, which are difficult to map in humans through radiological imaging, but nevertheless appear consistent with highly specialized modules for different sensory domains. The finding of shared activity patterns between pain, disgust and unfairness in left AI and mACC strongly supports the former interpretation, pointing to a supramodal representation of negative affect transcending sensory domains and event target. In this perspective, left AI/mACC might respond to the unpleasantness of events and therefore mediate signals of negative valence, or alternatively code for their arousal or perceptual salience^27^,^28^,^29^. Note, however, that arousal and salience also include positive affect. Future investigations will therefore need to test whether the effects identified here extend also to high-arousing positive stimuli.

Critically, a reliable part of our findings cannot be explained in terms of general, amodal features of aversive experiences. Instead, we found that activity patterns in right AI were specific for pain, disgust, unfairness, as well as the target of the experience. Indeed, overlapping fMRI activations are not necessarily diagnostic of common underlying representations^7^,^47^, but might reflect the presence of distinct (intermingled) neuronal populations related to modality- or target-specific representations. MVPA is well-suited to address these issues, as it investigates whether idiosyncratic spatial variations in the neural signal are shared or dissociated across experimental manipulations. Using this approach, Chikazoe *et al*.^47^ found modality-specific representations of unpleasantness in AI for gustatory and visual stimuli, as well as modality-independent representations in medial prefrontal regions. Our data extend these previous findings by demonstrating that AI codes for first-person pain and disgust through both modality-specific and modality-independent neural representations.

Taken together, our findings provide new and comprehensive insights into the neural processing of various aversive experiences. We show that the right AI contributes to modality-specific analysis of gustatory/nociceptive inputs, whereas the left AI and mACC might integrate the output of modality-specific computations into more abstract representations of these events. Future studies will need to further examine the differential role played by left AI and mACC in amodal processing of unpleasantness. For instance, it has been proposed that mACC might integrate negative affect, pain and cognitive control, presumably reflecting a key role played by this region in the selection of behavioural responses to unpleasant events^48^. Also the functional nature of the differential lateralization within AI for modality-specific and modality-independent processing remains to be elucidated. This could be partially determined by the lateralized nature of electrical stimuli (delivered on the left wrist), and consistent with suggestions that the insula contralateral to the stimulation site might process the sensory properties of a stimulus event, whereas the ipsilateral AI might code for its subjective meaning^25^. Evidence from experiments in which pain is delivered also on the right side of the body might support this claim^7^ (see Supplementary Note 1 and Supplementary Table 8). Alternatively, the right and left AI might exhibit distinctive contributions of sympathetic and parasympathetic systems, with the former involved in processing specific aversive events and the latter promoting shared regulatory/coping processes^49^. The observation of hemispheric differences in the disgust and fairness tasks (where stimuli were not lateralized) supports the latter interpretation. Overall, our results dovetail with seminal theoretical accounts of insula function, according to which AI and mACC mediate subjective feelings in terms of comprehensive representations of affective states that integrate multiple sources of information^8^,^19^,^45^.

AI and mACC were also engaged when participants observed a confederate undergoing pain or disgust. This finding converges with previous evidence demonstrating that these regions encode empathic responses elicited by various cues about others' negative states (abstract symbols, facial expressions, injured bodies and so on)^4^,^5^,^6^,^7^,^8^,^11^. The role of AI/mACC in both first-person and vicarious experiences has often been interpreted in favour of embodied models of social cognition, according to which the states of people are inferable by activating a representation of the same state in the observer^18^,^19^. In support of this view, we recently showed using MVPA that AI and mACC disclosed common activity patterns between felt and observed pain^7^. Yet, our previous results did not allow ascertaining whether the information shared between self and others concerned modality-specific or modality-independent features. The cross-modal effects observed in left AI and mACC strongly support a unique neuronal signature coding for aversive events regardless of the modality or recipient of the experience. Critically, however, we also found that activity evoked by vicarious empathic experiences in right AI was not shared with that elicited by first-person aversive states. This finding is consistent with previous evidence that human and monkey AI might process social emotions in partial dissociation from first-person pain^43^,^50^ or disgust^51^, and suggests the presence of target-specific neural activity complementing the shared representations.

Taken together these findings support embodied interpretation of left AI and mACC function, but at the same time circumscribe their reach. While neural responses encoding first-hand aversive experiences are re-activated when empathizing with others, these do not seem to contain modality-specific information related to the nociceptive or gustatory input anymore. A more suitable interpretation is that others' states trigger a neural representation of non-specific properties such as an unpleasant bodily experience, which was an inherent dimension of all events tested in our study^19^.

Finally, we show that neural signals in left AI and mACC related to the first-hand sensation of pain and disgust are in part shared with those associated with unfair treatments in the UG. Again no shared effects were observed in right AI, even though this region disclosed reliable activity patterns for unfairness. This suggests that right AI disclose modality-specific information, not only for pain and disgust, but also for the experience of unfairness, in parallel with an amodal representation of aversive events in left AI and mACC.

Although AI and mACC have frequently been implicated in unfair UG offers^16^,^36^,^37^,^38^,^39^, different accounts have been put forward to interpret the functional role of these regions in the bargaining game. Sanfey *et al*.^16^ suggested that AI activity might reflect an activation of disgust-specific representation in insular cortex, as a neural signature of ‘being disgusted by people's unfair behaviour'. This interpretation, in keeping with accounts suggesting how representations of unmoral behaviour are grounded on personal disgusting experiences^20^, is not supported by our data. Instead, our findings fit best the predictions of the wounded pride/spite model, according to which unfair behaviour elicits a broad negative emotional reaction in the observer^52^, with consequent physiological and neural responses.

Note, however, that previous studies have suggested a role of AI and mACC in the detection of equality violations in the UG^36^,^37^. Our data do not rule out a fairness-specific interpretation, since part of the predictive information about unfairness in right AI and mACC was independent from first-person experiences of pain or disgust. Hence, it is possible that AI/mACC process unfair offers in a multidimensional manner, taking into account both the equality of the offer and its emotional impact on individuals.

To our knowledge, our UG results are the first showing that a neural representation of first-hand pain/disgust is re-enacted, not only when empathizing with others, but also during other unpleasant social experiences. Interestingly, several studies also implicated the mACC during social pain, including aversive experiences caused by social exclusion, rejection or loss^53^,^54^. The role of the mACC in social pain has often been interpreted as a re-activation of signals of physical pain^54^,^55^ (but see Woo *et al*.^43^). Our data are in keeping with this interpretation, but only under the assumption that physical and social pain share a modality-independent representation of the unpleasantness of the experience. Future studies need to formally test this hypothesis by adopting the current decoding approach with the addition of social exclusion.

In conclusion, the present study aimed at clarifying the nature of information represented in AI and mACC, a network commonly engaged in first-hand pain, disgust or unfairness, but also when witnessing others undergoing the same aversive events. The recruitment of this network has often been interpreted in terms of domain-general affective processing, according to which these regions respond to common properties of the affective experience such as unpleasantness. Our data support domain-general coding in left AI/mACC while also promoting domain-specific processing, particularly in right AI that exhibited activity patterns uniquely distinctive for pain, disgust and unfairness, as well as for the person to whom the events were addressed. Taken together, these findings suggest that the AI–mACC network might subserve a comprehensive, multi-layer, representation of aversive experiences^25^,^44^,^45^, which takes into account both modality- and target-specific information and more general affective properties of the event.

## Methods

### Participants

Nineteen right-handed female volunteers (average age of 25.16±3.44 years, s.d.) took part in the experiment (see Supplementary Note 2 and Supplementary Fig. 9 for power analyses on independent pilot data). All participants were accompanied by a friend (12 females) who acted as confederate during the experimental session. Participants were free of psychiatric or neurological history and had normal or corrected-to-normal vision. They were all compensated according to both the overall experimental duration (8€ per hour) and participants' choices in the randomly selected trials of the UG task (see below). Written informed consent was obtained from all participants and confederates. The experiment was approved by the Ethical Committee at the faculty of Medicine of the University of Leipzig (Ethik-Kommission an der Medizinischen Fakultät der Universität Leipzig) and conducted according to the declaration of Helsinki.

### Experimental set-up

Participants and confederates took part in three experimental tasks implemented in blocks in alternating orders. The first task was a cue-based ‘empathy for pain' paradigm (pain task)^5^, in which participants either received an electrical (Ps or nPs) stimulation or were informed that their confederate was about to receive the same stimulation. In the second task, the cue-based empathy for pain paradigm was modified so that gustatory stimuli (Ds or nDs) were delivered either to participants or to the confederates (disgust task). The third task was the well-known UG paradigm in which participants were presented with monetary offers (unfair or moderately fair), which were addressed either to themselves or to the confederate^35^,^37^. Despite their differences, the employed experimental manipulations converged in exposing either participants or the confederates to aversive or neutral stimuli. This led, for each of the three tasks, to a 2 (UNPLEASANTNESS: aversive, neutral) × 2 (TARGET: self, other) factorial design, and to an overall of 12 conditions. All conditions were implemented while participants were lying supine in a MRI scanner and the confederates sat next to the scanner table in the MRI room.

The pain task was organized as follows. Consistently with earlier implementations of the ‘empathy for pain' task^5^, each trial started with the presentation of a coloured, arrow-shaped cue (2 s) that informed participants of the target and the unpleasantness of the upcoming stimulation (see Fig. 1a). Arrows pointing vertically downwards (↓) indicated stimulations directed to participants (self) while arrows pointing diagonally towards the confederate (↙) cued stimulations directed to the latter (other). The intensity of the cue's colour indicated the unpleasantness of the upcoming stimulation with light tones referring to non-noxious (neutral) stimulations and dark tones referring to noxious (aversive) stimulations. Different colours were used for self- and other-related trials. Colour codes changed across participants. Cues were followed by an electrical stimulation of 1 s (100 Hz) that was applied to participants' (or confederates') left wrist. Electrical stimulations were followed by a horizontal visual analogue rating scale (5 s) ranging from −3 (highly unpleasant) to +3 (highly pleasant). The direction of the scale was counterbalanced across trials to avoid motor preparation during the preceding stimulation periods. Participants marked the position of the scale corresponding to their judgment, by moving a slider (randomized initial position) with the index and middle fingers of their right hand operating a button box. Trials were separated by an inter-trial-interval ranging from 2.5 to 7.5 s (average 5 s) during which a white fixation cross was presented. Each of the four conditions of the pain task (pain stimuli delivered to self (Ps) or other (Po), plus corresponding nPs controls (nPs and nPo)) was repeated 16 times.

As for the pain task, in the disgust task as well participants were initially presented with cues (2 s) whose orientation (↓ or ↙) and colour informed about the target and the unpleasantness of the upcoming gustatory stimulation (see Fig. 1b). Colours were different from those used in pain trials and changed across participants. Cues were followed by 0.5 ml of liquid delivered on participants' (or confederates') tongues through the aid of plastic tubes. Participants were instructed to keep the liquid in their mouth and taste it for 4 s, until a ‘swallow' instruction (3 s) was presented on the screen. This ensured that movement artifacts in the neural signal due to swallowing occurred prevalently after the stimulation period. Subsequently, participants were asked to rate the experienced unpleasantness of the gustatory stimulus on the same scale used for the pain task (5 s). Finally, to minimize carry-over effects between subsequent trials, 2 ml of water were delivered via a separate tube to rinse (3 s), followed by another 3-s long swallowing period. Trials were separated by an interval of 2.5–7.5 s. Each of the four conditions of the disgust task (disgust stimuli to self (Ds) or other (Do), plus corresponding neutral controls (nDs and nDo)) was repeated 16 times. Participants didn't report coughing or other trouble due to the administration of gustatory stimuli.

Finally, we implemented an MRI-compatible version of the UG^16^. In each trial, participants faced an unknown proposer who made an offer on how to divide an initial endowment of 10€. The UG offers were addressed either to the self (standard UG) or to the other (third-party UG)^35^,^37^. In self-related trials of the UG, participants were told that if they accepted the offer, the money would be divided between them and the proposer accordingly, whereas if they rejected it, both they and the proposer would not get any money. In other-related trials, participants responded to the offers on behalf of the confederate without any economical consequence for themselves. Thus, if participants accepted, the money would be divided between the confederate and the proposer while both got nothing if participants rejected the offer. Proposers were described as 96 university students from a different city (Geneva, Switzerland), which previously made one single UG offer, thus leading to 96 independent 1-shot interactions with the participants (recruited in Leipzig, Germany). Regardless of the cover story, participants faced offers that were defined *a priori* by the experimenters, and which ranged from extremely unfair (the proposer offered 1 or 2€ out of 10€ (1:9, 2:8) and wished to keep the remaining), to moderately unfair (3:7, 4:6) and extremely fair (5:5, 6:4). Participants and confederates knew that 2 of the 96 bargaining trials (one self- and one other-related) would be randomly selected at the end of the experimental session and implemented according to the participants' choices. Self-reports obtained subsequent to scanning confirmed that participants believed that the proposers were genuinely human (average of 4.16±1.21 s.d. on a scale ranging from 1 (I did not believe at all) to 5 (I absolutely believed)).

Reminiscently to the pain and disgust tasks, UG trials were introduced by a cue (2 s) whose orientation (↓ or ↙) and colour informed about the target of the upcoming offer. Unlike for the other tasks, UG cues were not informative of the alleged unpleasantness of the upcoming event (same brightness for all six implemented offers). To increase the plausibility of the cover story, arrows were presented together with a photo taken from the NimStim face database^56^ (48 males, 48 females displaying neutral facial expression; age ranging from 18 to 34 years). Cues were followed by an offer screen (4 s), which was schematically represented as two piles of coins (left for proposer, right for participant/confederate). The proposed split was also explicitly reported beneath the piles. Participants responded by pressing one of two buttons assigned to ‘accept' or ‘reject' as displayed at the lower portion of the screen. Following their response, participants were asked to rate the experienced unpleasantness on the same scale used for the other tasks (5 s). Trials were separated by an interval of 2.5–7.5 s. Overall, the UG comprehended 48 self- and 48 other-related trials: 32 of these conveyed 1:9 and 4:6 offers (16 repetitions each), whereas the remaining 16 trials conveyed 2:8, 3:7, 5:5 and 6:4 offers (4 repetitions each). Based on individuals' unpleasantness ratings, these trials were then sorted into 4 subgroups of 16 trials each (unfair offer to self or to other (U), plus corresponding midfair controls (M)) which were most closely matched for unpleasantness with homologous conditions in the pain and disgust tasks. Please notice that, for the purpose of the present study, we are focusing on UG effects obtained when merging self-related and other-related trials together Supplementary Fig. 2 report effects associated with each target separately.

### Procedure and apparatus

Prior to scanning, participants and confederates underwent brief thresholding sessions to identify electrical and gustatory stimuli that elicited comparable levels of unpleasantness^34^ (see below). The scanning session was divided into four functional runs. Each run was in turn divided into three blocks, corresponding to the three tasks. A text string (3 s) informed the participant of the task in the upcoming block. The order of blocks was counterbalanced across runs and across participants. During the four functional runs, confederates were placed in a chair next to the scanner with their eyes masked, and were cued for tasks and trial conditions using different audible beep signals presented via headphones. Subsequent to the four functional runs, we obtained functional resting-state data and a high-resolution anatomical scan for each participant. During the resting-state session, participants were instructed to keep their eyes open, to fixate a centrally presented white cross against a black background and to think of nothing in particular. Following the scanning session, participants and confederates were debriefed. Each experimental session took ∼2.5 h.

Electrical stimulations to the left wrist were delivered via MRI-compatible, gold-based electrodes attached to a DS7A Constant Current Stimulator (Digitimer Ltd). Liquids were automatically presented via separate plastic tubes (diameter of 1.5 mm) that rested at the tip of the tongue using a custom-build computer-controlled pump-system. Tubes were fixated to the head coil with a plastic holder. Visual stimuli were projected on a screen (about 19° × 14° of visual angle) placed inside the scanner bore. Key-presses were recorded on an MRI-compatible response button box. The task was programmed using Presentation 14 (Neurobehavioral Systems) software.

### Pain thresholding

The amplitudes of noxious and non-noxious stimulations varied on an individual basis and were selected, for participants and confederates separately, prior to the scanning session through a multiple-random-staircase approach^57^. Computerized pain thresholding was realized using four staircases of single electrical stimulations (ranging from 0 mA to a maximum of 2.5 mA). Each electrical stimulus (1 s) was announced by a countdown (‘Stimulus in 3/2/1', duration of 3 s), and followed by a VAS ranging from 1 (barely detectable) to 10 (strongest imaginable pain) through which subjects rated their subjective pain experience. Subjects had also the option to select a value of 0 (presented left to the scale) to report imperceptible stimuli. Ratings were self-paced and followed, after 200 ms, by a new electrical stimulation from a different (randomly selected) staircase. Two staircases aimed at identifying the subject-specific threshold intensity of a noxious stimulus, whereas the remaining two aimed at identifying the intensity of the non-noxious (but yet detectable) stimulus.

The staircases started with electrical intensities randomly ranging from 0.6 to 0.8 mA (noxious stimulus) or from 0.25 to 0.45 mA (non-noxious stimulus). Subsequently, intensities were systematically increased or decreased by increments of 0.08 mA until the experienced pain exceeded or fell below a predefined subjective pain value. In that case, the staircase was reversed until the subjective pain value was met again (turning points). Each staircase stopped once collecting eight turning points (four below and four above the subjective pain value). Noxious stimuli were calculated as the average intensity (mA) associated with 14 turning points (the last seven of two staircases) around a subjective pain value of 7.5 (out of 10). Non-noxious (but detectable) stimuli were calculated as the average intensity associated with 14 turning points around a subjective pain value of 1.5 (out of 10). The subjective pain value used to identify noxious and non-noxious stimuli was unknown to the subjects.

The noxious and non-noxious intensities identified through the staircase approach were then re-tested in a second self-paced, computerized task that required subjects to judge the subjective unpleasantness of stimuli on a VAS similar to the one used in the experimental session. The pain stimuli were expected to elicit subjective unpleasantness levels comparable to those triggered by the gustatory stimuli, that is between −2 and −2.5 for noxious stimuli (on a scale from −3 to 3) and between −0.5 and 0 for non-noxious stimuli. If this was not the case, the intensity of the stimuli was further adjusted. Overall, in 19 participants undergoing fMRI, the pain thresholding procedure led to an average noxious stimulus of 1.2 mA (±0.1, s.d.), and to an average non-noxious stimulus of 0.4 mA (±0.03).

### Disgust thresholding

Disgusting and neutral liquids were selected prior to the experimental session to identify, for participants and confederates separately, two gustatory stimulations whose unpleasantness matched the electrical stimuli used for the pain task. In keeping with earlier neuroimaging studies^6^,^58^,^59^ and pilot data collected on independent subjects (see Supplementary Note 1), negative stimuli were chosen from three salt solutions (NaCl) of different degrees of dilution (0.1, 0.5, 1 mol) and three concentrations of quinine solution (0.25, 1, 10 mM). Neutral gustatory stimuli consisted of four solutions with the main ionic components of saliva (25 mM KCl and 2.5 mM NaHCO_3_), diluted with various amounts of distilled water (20, 40, 60 and 80%).

The thresholding procedure was organized as follows. On each trial, 0.5 ml of a solution were administered (via syringes without needle) to the subject who evaluated the experienced unpleasantness on a VAS similar to the one used during scanning. Three kinds of substance (salt, quinine, saliva) were administered separately, with the order of substances and concentrations randomized across individuals. For each subject, we selected one negative stimulus associated with an unpleasantness range from −2.5 to −2 (on a scale from −3 to 3) and one neutral stimulus with a rating from −0.5 to 0. Furthermore, subjects were screened for their taste sensitivity concerning the gustatory stimuli using a labelled magnitude scale with a continuous rating from 0 (barely detectable) to 100 (strongest imaginable)^60^. The subjective taste intensities for the chosen negative solutions were found to be within the range of 30–75, consistent with earlier studies^59^.

### fMRI data aquisition

Functional imaging was performed on a 3T Verio scanner (Siemens Medical Systems, Erlangen) equipped with a 12-channel head coil. An echoplanar imaging sequence (repetition time (TR)=2 s, echo time (TE)=30 ms, flip angle=90°, 3 × 3 × 3 mm, 1 mm interslice gap, matrix size 64 × 64, 30 slices tilted at ∼30° from axial orientation) was used to obtain T2*-weighted functional images. Functional image acquisition was realized in 4 functional runs, each consisting of 524 volumes (∼18 min, total of ∼72 min). Functional runs were divided by breaks of ∼2 min, where no functional images were obtained. In addition, we collected task-free fMRI time series, consisting of 200 volumes acquired over ∼7 min using the same sequence as the task-related fMRI. Subsequent to the functional image acquisition, a T1-weighted high-resolution anatomical image was obtained using a MPRAGE sequence (TR=2.3 s, TE=2.98 ms, flip angle=9°, 1 × 1 × 1 mm, matrix size 240 × 256, 176 sagittal slices, ipat=2) and a 32-channel head coil (∼5 min).

### fMRI data processing

The acquired images were processed using the SPM8 software (http://www.fil.ion.ucl.ac.uk/spm/). For each subject and for each functional run, the first three volumes were discarded. The remaining images were corrected for head movement between scans by an affine registration. In none of the subjects, head movements within each functional run exceeded 3 mm per degree, confirming that electrical stimulation and swallowing of gustatory stimuli did not result in unusual movement artifacts in the present study. Subsequently, to compensate for time-acquisition delays, each temporal slice was temporally realigned to the 15th (of 30) reference slice through interpolation. The resulting functional images were aligned to the T1-weighted anatomical image through rigid-body registration, and smoothed using a 6 mm full-width at half-maximum (FWHM) Gaussian kernel.

We analysed the preprocessed data from our task-positive sessions using the general linear model (GLM) framework implemented in SPM. For each participant and in each functional run, the onsets of each trial were modelled independently, yielding trial-wise parameter estimates of the conditions-of-interest^7^. In line with previous implementations of the pain task^5^, for the pain and disgust trials we considered relevant the neural activity associated with both the presentation of the cue and the subsequent stimulation, thus leading to epochs which were 3-s (pain) and 6-s (disgust) long (see Fig. 1a,b). For the UG, in which the cue was not informative of the unpleasantness of the upcoming event, we modelled the epochs in which the monetary offers were made (4 s) (see Fig. 1c). In addition, we also modelled epochs in which the cues were presented as regressors of no interest (2 s). All the resulting vectors were convolved with a canonical haemodynamic response function as implemented in SPM. Movement parameters were included as covariates of no interest. Low-frequency signal drifts were filtered using a cutoff period of 128 s. For each subject, this GLM lead to as many parameter estimate (*β*s) images as experimental trials were employed in the study. These images were then fed to multivoxel pattern analysis (MVPA) routines.

### Multivoxel pattern analysis

We run classification-based MVPA to assess the similarity between the neural representations of our experimental conditions. The analysis was conducted by first defining a ROI and then by extracting, from each of its constitutive coordinates, the parameters estimates (*β*s) associated with each experimental trial. Furthermore, for each individual subject, and for each condition, response patterns were mean centred through *z*-transformation to ensure that the MVPA analysis would not be biased by differences in the average ROI activity across conditions^61^. Data were then fed into a linear kernel SVM classifier (using a fixed regularization parameter, *C*=1), which operates by finding an optimal linear decision boundary (hyperplane) that separates experimental classes with maximum margin. New data (not used to define the decision boundary) are classified according to which side of the hyperplane they fall onto^62^. Signal detection methods were used to compute *d*^63^ as a measure of the sensitivity of the hyperplane to detect, in new data, the occurrence of one aversive condition. Classification analysis was performed using the LIBSVM 3.18 software^40^.

Statistical validation of the average *d′* obtained at the group level was achieved through rigorous non-parametric permutation techniques, following guidelines on statistical assessment of decoding accuracies^64^. In particular, for each classification analysis, we created 1,000 permutation schemes aimed at breaking the relationship between the data and the condition labels. Each of these permutations was applied to the data of all subjects and all ROIs, which were then fed to the same classification routines used for the original data set. All the group-wise *d′* values obtained through permuted data sets were used to estimate critical cutoffs, above which an effect could be considered significantly higher than null. More specifically, to assess significant (albeit uncorrected) effects within each ROI, we used as cutoff the 95th percentile of the permutation distribution of 1,000 group-wise *d′*s obtained from the data of that ROI. Following previous implementations of *P* value adjustment in permutation-based multiple testing^65^, we computed the permutation distribution of the 1,000 maximal group-wise *d′*s over all ROIs to provide corrections for multiple comparisons. The 95th percentile of this ‘maximal group-wise *d′*s' distribution represents a reliable cutoff for *d*′s that are unlikely (*P*<0.05) to be achieved in any ROI under the null hypothesis. Similar permutation analyses were carried out to assess whether the *d′* difference between two conditions (diff-*d′*), or the value from standardized statistical tests (for example, F test in a repeated measure ANOVA testing *d′* differences across ROIs), were significantly higher-than-chance.

As a manipulation check, we first assessed the ability of a linear SVM classifier to discriminate between data associated with each aversive condition from the data associated with its tailored neutral control (for example, Ps versus nPs). Specifically, we employed leave-one-pair-out cross-validation, in which the 16 aversive and 16 neutral trials were randomly paired together. We then tested, in 16 independent folds, whether the data from each pair could be correctly classified by a hyperplane estimated on the remaining 15 pairs. For each participant, we estimated *d′* from the classified trials of all the implemented folds. This analysis provides an estimate of the information about unpleasantness present in each ROI, separately for each modality and target, regardless of whether or not it is shared with other experimental manipulations (within-task classification).

Subsequently, we ran cross-modal classification that assessed whether a classifier trained to detect unpleasantness in one specific task (for example, Ps) could discriminate unpleasantness from another modality (for example, Ds). The analysis was conducted through 2 independent folds: we first trained a SVM on the 32 trials associated with Ps and nPs, and then tested the ability of the estimated hyperplane to classify the 32 trials associated with Ds and nDs. In the second fold, we trained a SVM on the trials associated with Ds and nDs, and then tested the estimated hyperplane on the trials associated with Ps and nPs. *d′* values were then estimated from the classified trials from both folds. Reliable cross-modal classifications (Ps↔Ds) can be interpreted consistently with Ps and Ds triggering (each relatively to its tailored control) a partly similar response pattern. A similar approach was implemented to test for shared activity patterns across different targets (cross-target classification) (for example, Ps↔Po), or for cross-modal effects in different targets (Ps↔Do).

The MVPA analyses described were first run on *a priori* defined ROIs of AI and mACC (see below and Results). Furthermore, we implemented a searchlight-decoding approach that does not rely on *a priori* assumptions about informative brain region but searches for predictive information throughout the whole brain^7^,^32^,^33^,^41^,^42^: for each coordinate of the individual native brain image, a spherical volume-of-interest surrounding the coordinate was defined (5 voxels radius, 81 voxels total). The parameter estimates of all voxels in the sphere were extracted and a classification procedure similar to the ROI-based MPVA was implemented. Resulting *d′* values were assigned to the centre voxel of the sphere and the procedure was repeated for the next voxel. For each participant, this led to *d′*-maps which were then spatially normalized to the Montreal Neurological Institute (MNI) single-subject template, using the deformation field obtained during the normalization of the T1-weigthed anatomical image. The resulting normalized images were smoothed using a 6 mm FWHM Gaussian kernel and fed into group level analyses using one-sample *t*-tests to search for regions in which the group-wise *d′*>0. Voxels were identified as significant only if they passed an extent threshold corresponding to *P*<0.05 corrected for multiple comparison, with an underlying height threshold of at least *t*_(18)_=3.61, corresponding to *P*<0.001 (uncorrected). We used as extent threshold the 95th percentile of the distribution of the largest cluster obtained through 1,000 replications of the same analysis on permuted data sets. Second-level *t*-tests were performed using the SnPM toolbox of SPM (http://warwick.ac.uk/snpm).

### Resting-state analysis and ROI definition

We used group-independent component analysis (ICA) toolbox GIFT 3.0a (http://icatb.sourceforge.net/) to estimate the ICs corresponding to functional networks, based on fMRI data from the resting-state sessions. Group ICA, which seeks ICs for the group data instead of estimating networks separately for each individual, was chosen to avoid the ambiguity arising from combining different individual networks obtained by separate estimations. For this reason, the resting-state data from individual subjects were fed to the same preprocessing routines of the task-positive sessions, with the exception than functional images were first warped in the common stereotaxic MNI space before being smoothed with a 6 mm FWHM Gaussian kernel. The minimum-description-length algorithm^66^ run on the data estimated the number of sources to be 28. Then, subjects' data were reduced to a lower dimensionality by using a two-stage principal component analysis (one at an individual level and one at a group level). Then, data from all subjects/sessions were concatenated and independent group components were estimated using the Infomax approach^67^. The ICASSO method^68^ was used to assess the reliability of the networks by running the algorithm 30 times, each time with different initial conditions and bootstrap resampled data sets.

Figure 2a shows the IC implicating the bilateral AI and mACC, and therefore corresponding to the relevant affective/salience network described in previous studies^28^. Representations of AI and mACC in each individual native space were obtained through an anatomically constrained functional approach. Back reconstruction of the IC and time courses was done with the GICA3 algorithm^69^. The resulting individual maps were masked by excluding coordinates outside of the insular cortex (for AI) or the cingulate cortex (for mACC) as defined by the AAL atlas^70^. The resulting masked image was then warped into the individual native space using a deformation field inverse to the one estimated during normalization. We finally selected, as features for the MVPA analysis, those 81 coordinates (same number as in the whole-brain searchlight approach) in the native space that exhibited the largest contribution in the IC-of-interest. For each participant, three individual ROIs were created, corresponding to right and left AI and mACC. Supplementary Figure 8 depicts these selected coordinates in six different representative brains.

## Additional information

**How to cite this article:** Corradi-Dell'Acqua, C. *et al*. Cross-modal representations of first-hand and vicarious pain, disgust and fairness in insular and cingulate cortex. *Nat. Commun.* 7:10904 doi: 10.1038/ncomms10904 (2016).

## Supplementary Material

Supplementary Information Supplementary Figures 1-9, Supplementary Tables 1-8, Supplementary Notes 1-2 and Supplementary References

## Figures and Tables

**Table 1:** Within-task pattern analysis.

	*d'* (corrected cutoff)
*Left AI*	
*Ps* versus *nPs*	**0.98** (0.32)**
*Ds* versus *nDs*	**0.61** (0.29)**
*Po* versus *nPo*	**0.49** (0.27)**
*Do* versus *nDo*	**0.24** (0.26)*
*Us* versus *Ms*	**0.30** (0.28)**
*Uo* versus *Mo*	0.19 (0.20)^†^
	
*Right AI*	
*Ps* versus *nPs*	**1.29** (0.32)**
*Ds* versus *nDs*	**0.49** (0.29)**
*Po* versus *nPo*	**0.36** (0.27)**
*Do* versus *nDo*	**0.40** (0.26)**
*Us* versus *Ms*	**0.33** (0.28)**
*Uo* versus *Mo*	**0.38** (0.28)**
	
*mACC*	
*Ps* versus *nPs*	**1.61** (0.32)**
*Ds* versus *nDs*	**0.65** (0.29)**
*Po* versus *nPo*	**0.46** (0.27)**
*Do* versus *nDo*	**0.53** (0.26)**
*Us* versus *Ms*	**0.77** (0.28)**
*Uo* versus *Mo*	**0.76** (0.28)**
	
	***F* (cutoff)**
*Rep. Meas. ANOVA*	
*Ps* versus *nPs*	1.96 (3.54)
*Ds* versus *nDs*	0.62 (3.00)
*Po* versus *nPo*	0.21 (3.35)
*Do* versus *nDo*	0.91 (3.21)
*Us* versus *Ms*	**3.18** (3.12)*
*Uo* versus *Mo*	**4.57** (3.25)*

AI, anterior insula; ANOVA, analysis of variance; mACC, mid-anterior cingulate cortex; MVPA, multivoxel pattern analysis; ROI, region-of-interest.

**P<0.05, *P<0.05 under uncorrected cutoff, ^†^*P*=0.06.

For each ROI, MVPA was conducted to assess the presence of multivoxel activity patterns discriminative of one negative condition relative to its tailored neutral control. Detection of negative events was measured as *d′* coefficients. Higher-than-chance detections are reported when *d′* values exceeded the 95th percentile of null *d′*-distribution (cutoff), obtained with 1,000 replications of the analysis on permuted data sets (see Methods section). F values (and corresponding permutation-based cutoffs) from repeated measure ANOVAs testing for ROI differences are also reported. Significant effects are highlighted with corresponding *P* values in the table.

**Table 2:** Cross-modal and cross-target pattern analysis.

	*d'* (corrected cutoff)
*Left AI*	
*Ps*↔*Ds*	**0.31** (0.18)**
*Ps*↔*Po*	**0.21** (0.20)**
*Ds*↔*Do*	**0.19** (0.19)*
*Ps*↔*Do*	**0.30** (0.20)**
*Ds*↔*Po*	**0.18** (0.20)*
*Ps*↔*U*	**0.27** (0.17)**
*Ds*↔*U*	**0.32** (0.16)**
	
*Right AI*	
*Ps*↔*Ds*	0.02 (0.18)
*Ps*↔*Po*	0.11 (0.20)
*Ds*↔*Do*	0.12 (0.19)
*Ps*↔*Do*	0.10 (0.20)
*Ds*↔*Po*	**0.23** (0.20)**
*Ps*↔*U*	0.03 (0.17)
*Ds*↔*U*	0.03 (0.16)
	
*mACC*	
*Ps*↔*Ds*	**0.63** (0.18)**
*Ps*↔*Po*	**0.35** (0.20)**
*Ds*↔*Do*	**0.25** (0.19)**
*Ps*↔*Do*	**0.59** (0.20)**
*Ds*↔*Po*	**0.43** (0.20)**
*Ps*↔*U*	**0.44** (0.17)**
*Ds*↔*U*	**0.24** (0.16)**
	
	***F* (cutoff)**
*Rep. Meas. ANOVA*	
*Ps*↔*Ds*	**6.77** (3.39)*
*Ps*↔*Po*	1.66 (3.14)
*Ds*↔*Do*	0.36 (3.06)
*Ps*↔*Do*	**4.08** (3.21)*
*Ds*↔*Po*	1.54 (3.35)
*Ps*↔*U*	**4.56** (3.53)*
*Ds*↔*U*	**2.87** (3.16)^†^

AI, anterior insula; ANOVA, analysis of variance; mACC, mid-anterior cingulate cortex; MVPA, multivoxel pattern analysis; ROI, region-of-interest.

**P<0.05, *P<0.05 under uncorrected cutoff; ^†^F test *P* (one-tailed)=0.05.

For each ROI, MVPA was conducted to assess whether multivoxel activity patterns discriminative of one specific unpleasant experience (for example, self-pain) could detect unpleasantness of another modality/target. Detection of negative events was measured as *d′* coefficients. Higher than chance detections are reported when *d′* values exceeded the 95th percentile of the null *d′*-distribution (cutoff), obtained with 1,000 replications of the analysis on permuted data sets (see Methods). F values (and corresponding permutation-based cutoffs) from repeated measure ANOVAs testing for differences in *d′*s across ROI are also reported. Significant effects are highlighted with corresponding *P* values.
