# Supplementary material for: Cross-modal representations of first-hand and vicarious pain, disgust and fairness in insular and cingulate cortex
Source: Nat Commun. 2016 Mar 18;7:10904. doi: 10.1038/ncomms10904 (PMC4802033; doi:10.1038/ncomms10904)
Supplement: Supplementary Information — Supplementary Figures 1-9, Supplementary Tables 1-8, Supplementary Notes 1-2 and Supplementary References [file ncomms10904-s1.pdf]

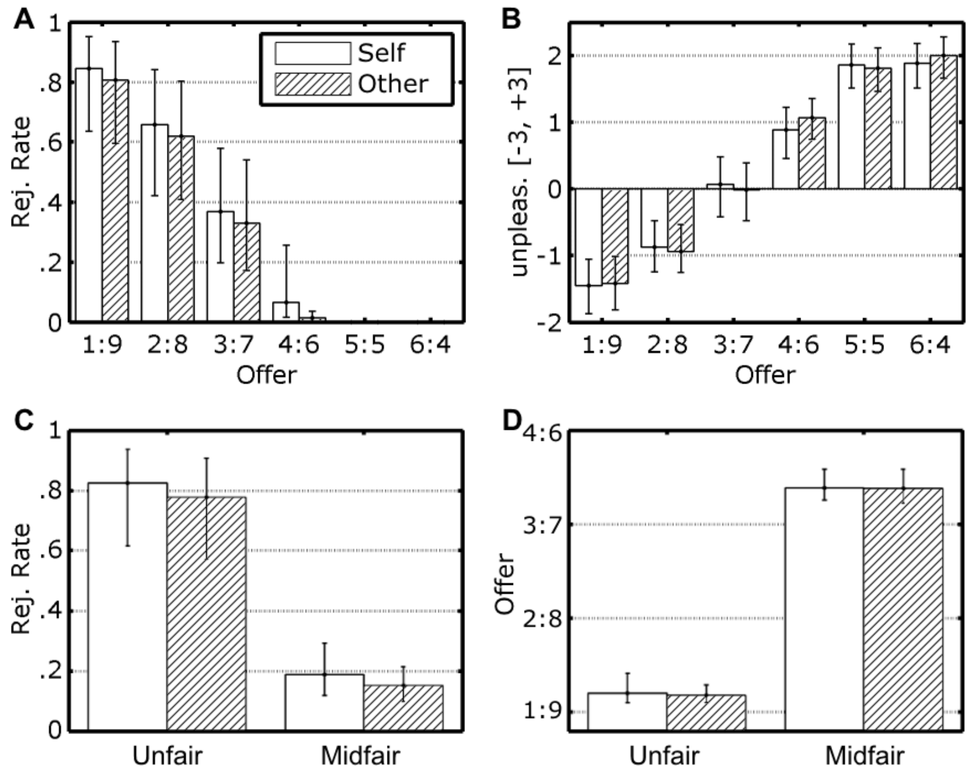


**Supplementary Figure 1. Detailed behavioral results of the Ultimatum Game Task.** (**A**) Average rejection rates and (**B**) median unpleasantness ratings associated with different kinds of offers. Empty bars refer to trials in which the offer was addressed to the participant, whereas striped bars refer to trials in which the offer was addressed to the confederate. Error bars refer to bootstrap-based 95% confidence intervals. (**C**) Average rejection rates and (**D**) monetary amount offered by the proposer, in those trials rated as the most unpleasant (16 trials addressed to the self [empty bars] and 16 trials addressed to the other [striped bars]), and the most neutral (16 to the self and 16 to the other), in each participant. The most unpleasant trials were those in which the proposer was highly unfair (1 or 2 € offered out of 10), and therefore globally labeled as unfair. Neutral trials were those in which the proposer made a moderately (un)fair but more balanced offer (3 or 4 € out of 10), globally labeled as midfair. Unfair and midfair offers were those subsequently used in our main analyses.

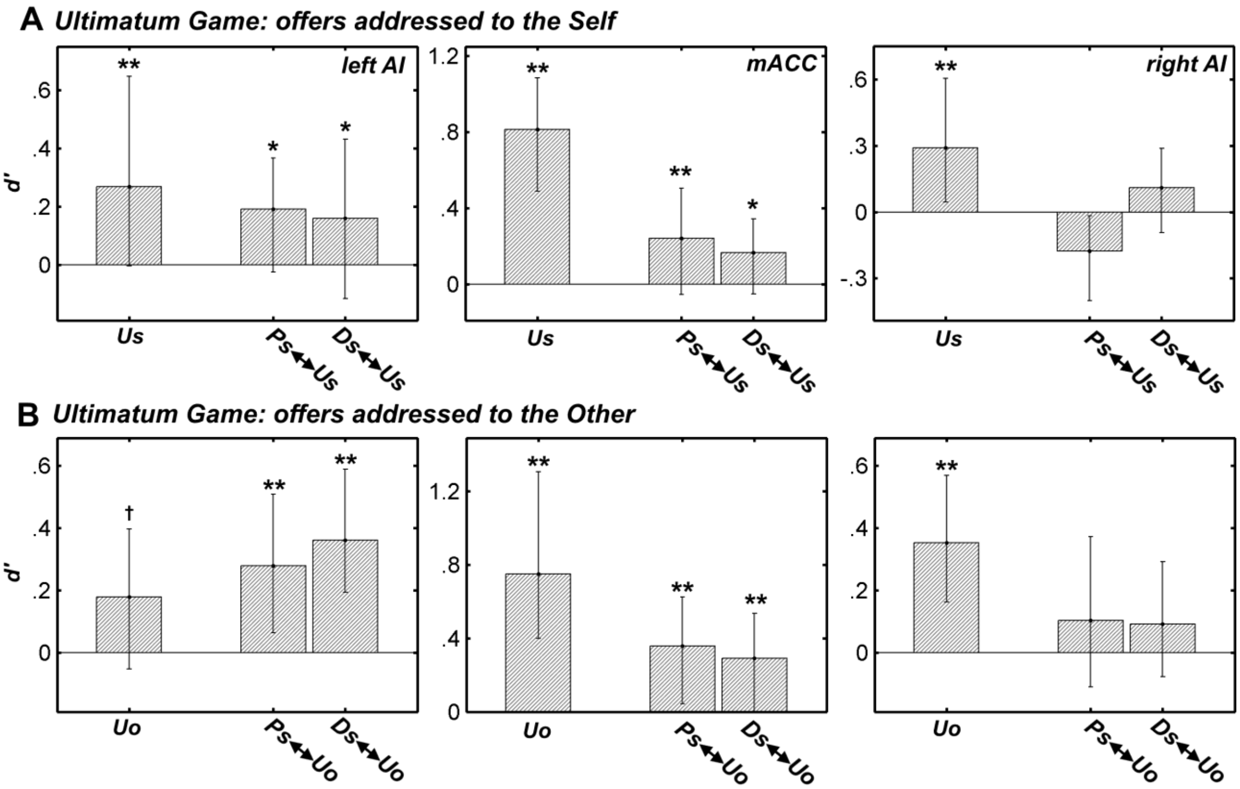


**Supplementary Figure 2. Detailed ROI analysis of the Ultimatum Game Task.** Bar-plots display *d'* values representing the ability of a linear kernel SVM classifier to detect activity patterns characteristic for (**A**) first-person (Self [s]) or (**B**) vicarious (Other [o]) experience of unfairness [U] in ROIs in left AI, mACC and right AI. Us and Uo refer to within-task classifications of unfairness for each target. Ps ↔ Us, Ds ↔ Us, Ps ↔ Uo, Ds ↔ Uo, refers to cross-modal classifications between both self-related aversive events (self-related Pain, [Ps], self-related Disgust [Ds]). The significance of permutation tests comparing *d'* values against chance (or against values from other conditions) are also reported. ** $p < 0.05$ corrected for multiple comparisons for the three ROIs; * $p < 0.05$ uncorrected. Error bars refer to bootstrap-based 95% confidence intervals.

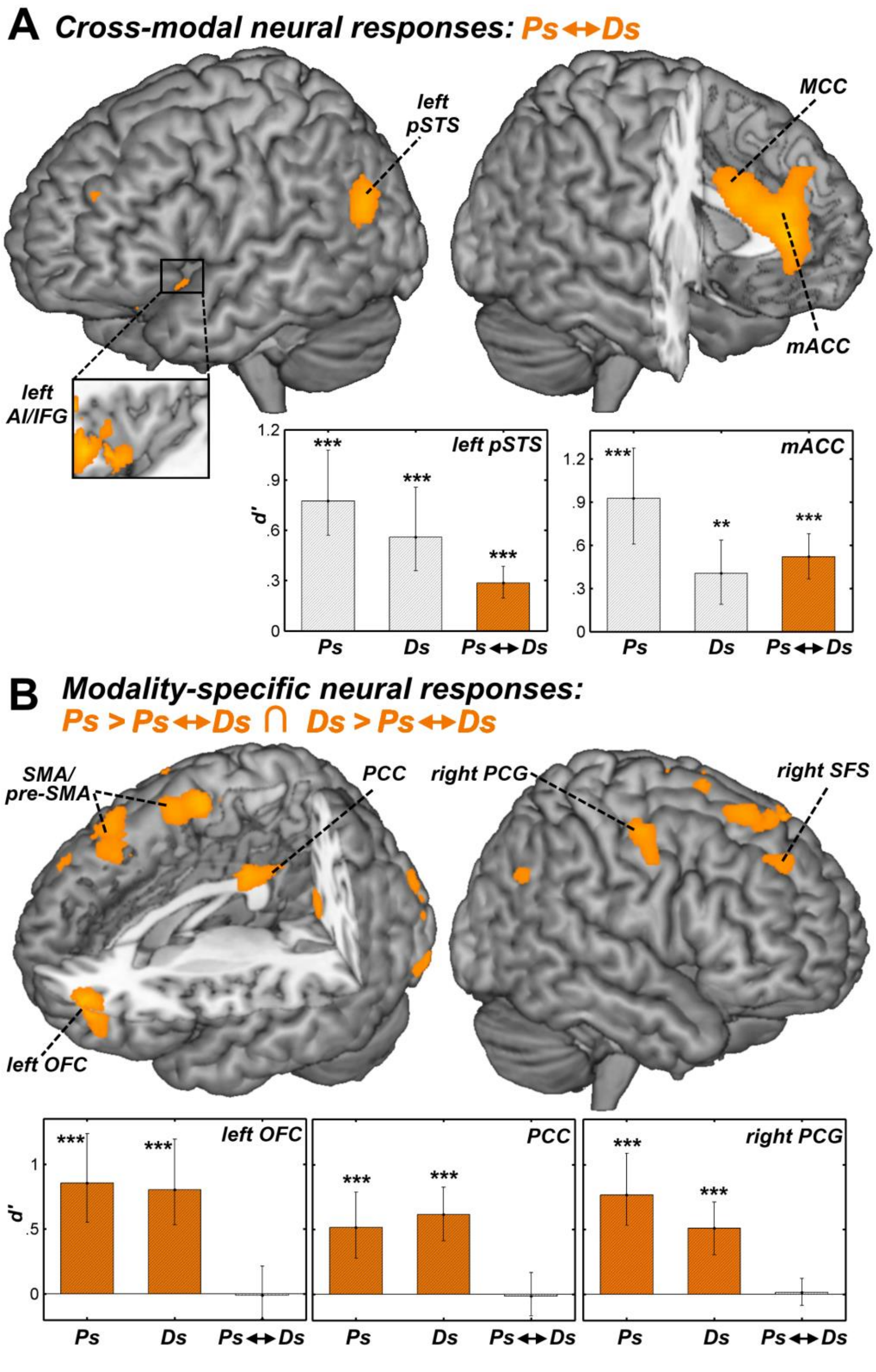


**Supplementary Figure 3. Shared and dissociated representations of first-hand pain and disgust.** (**A**) Whole brain maps displaying regions associated with cross-modal classification of first-person pain and disgust (Ps ⟷ Ds). All regions (listed extensively in Supplementary Table 2) survive rigorous permutation-based correction for multiple comparisons at the cluster level.

(**B**) Whole brain maps displaying regions exhibiting activity patterns sensitive to both first-person pain and disgust in a modality-specific fashion (Ps > Ps ↔ Ds ∩ Ds > Ps ↔ Ds). IFG: inferior frontal gyrus; pSTS: posterior superior temporal sulcus; PCC: the posterior cingulate cortex; PCG: the postcentral gyrus; OFC: lateral orbitofrontal cortex; SMA: supplementary motor area; SFS: and superior frontal sulcus. D' values extracted from the outlined regions are plotted in bar graphs. Orange bars refer to statistical classification tests for each region. The significance of *t*-tests that compared *d'* values against chance level are also reported. *** $p < 0.001$; ** $p < 0.01$. Error bars refer to bootstrap-based 95% confidence intervals.

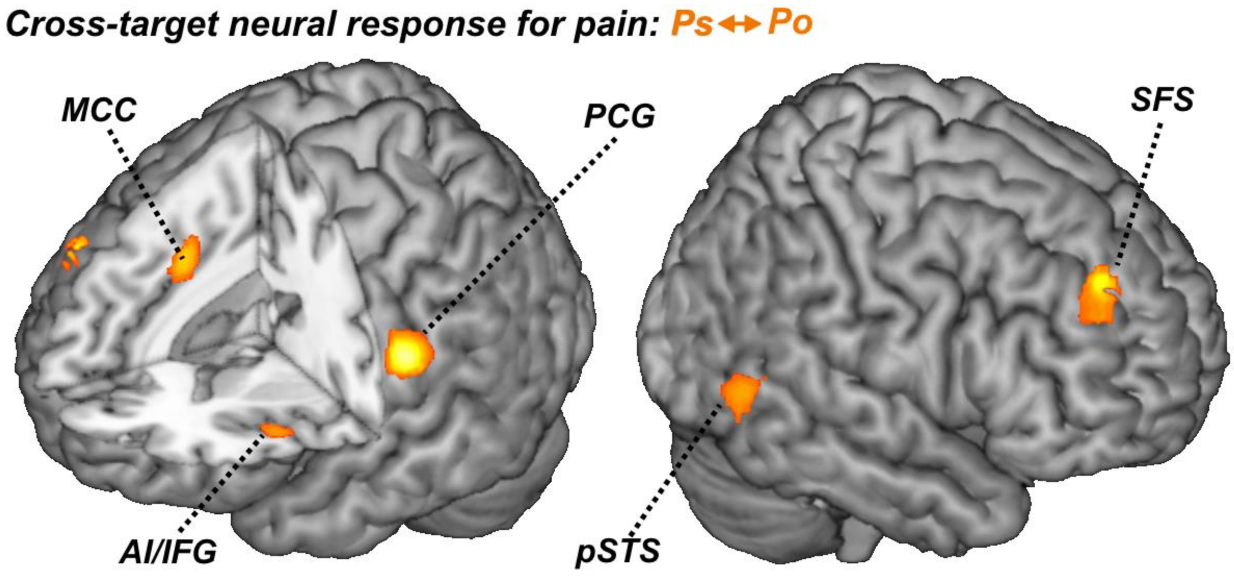


**Supplementary Figure 4. Shared representations of first-hand and vicarious pain.** Whole brain maps displaying regions associated with cross-target classification of first-person and vicarious pain (Ps ↔ Po). All regions are listed extensively in Supplementary Table 3.

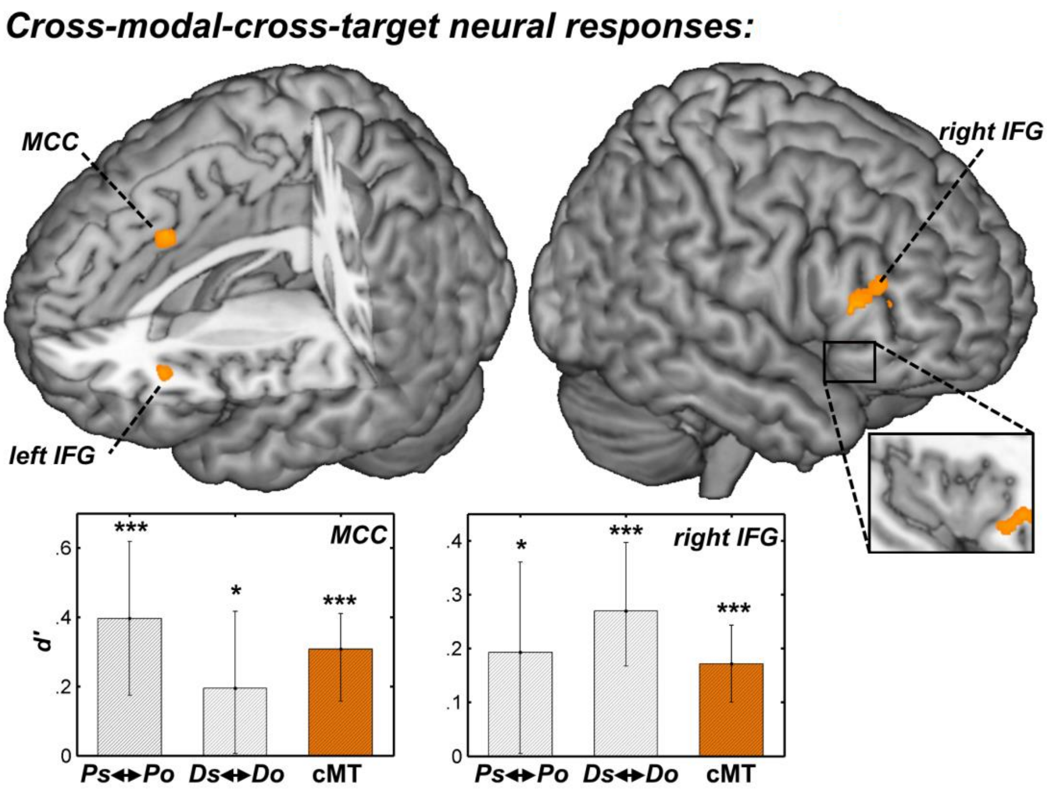


**Supplementary Figure 5. Shared patterns across modalities and targets.** Whole brain maps displaying regions associated with a reliable cross-modal responses for pain and disgust across different targets (Ps ↔ Do and Ds ↔ Po). All regions (listed extensively in Supplementary Table 5) survive rigorous permutation-based correction for multiple comparisons at the cluster level. D' values extracted from the outlined regions are plotted in bar graphs. Orange bars refer to statistical classification tests for each region. The significance of *t*-tests that compared *d'* values against chance level are also reported. *** $p < 0.001$; * $p < 0.05$. Error bars refer to bootstrap-based 95% confidence intervals.

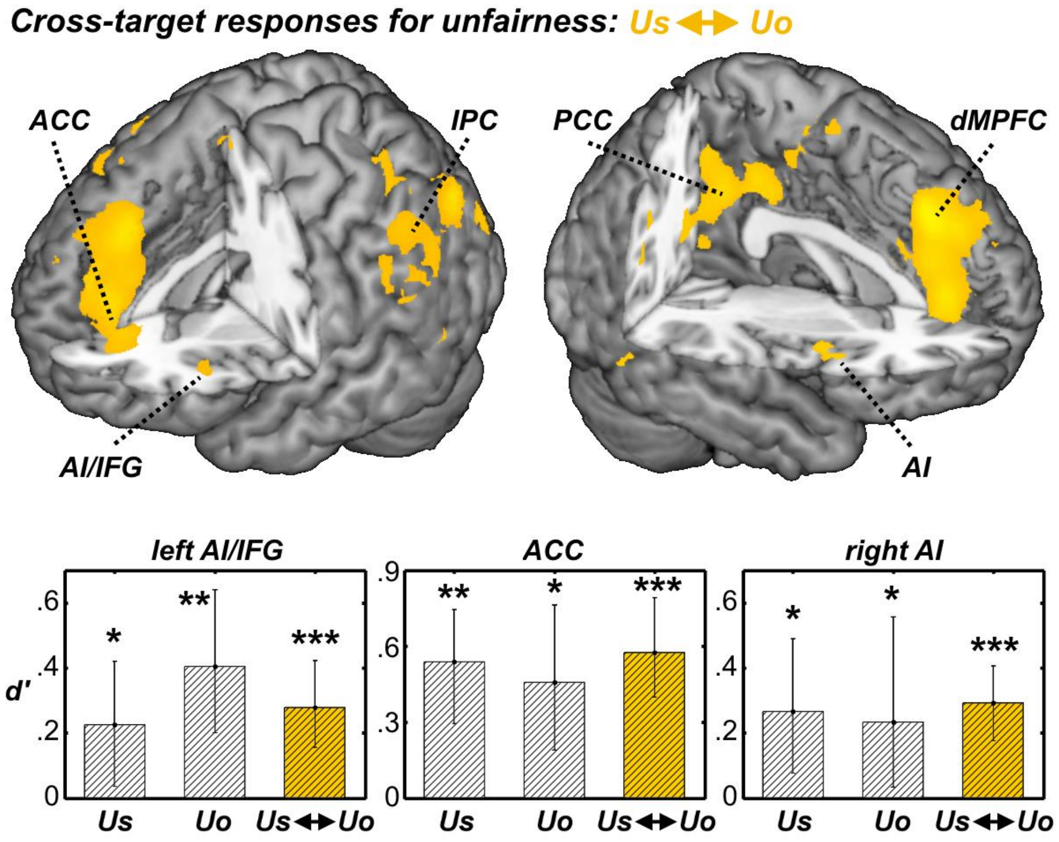


**Supplementary Figure 6. Shared representations of first-hand and vicarious unfairness.** Whole brain maps displaying regions associated with cross-target classification of first-person and vicarious unfairness (Us ↔ Uo). All regions are listed extensively in Supplementary Table 7. D' values extracted from the outlined regions are plotted in bar graphs. Yellow bars refer to statistical classification tests for each region. ACC: anterior cingulate cortex; dMPFC: dorsomedial preronal cortex; IPC: inferior parietal cortex. The significance of *t*-tests that compared *d'* values against chance level are also reported. *** $p < 0.001$; ** $p < 0.01$; * $p < 0.05$. Error bars refer to bootstrap-based 95% confidence intervals.

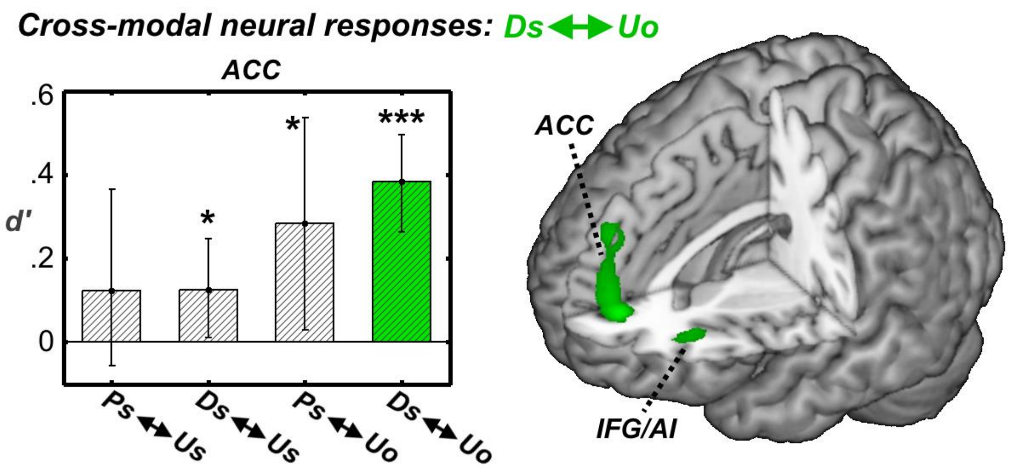


**Supplementary Figure 7. Shared representations of first-hand disgust and vicarious unfairness.** Whole brain maps displaying regions associated with cross-modal cross-target classification of first-person disgust and vicarious unfairness (Ds ↔ Uo). All regions are listed extensively in Supplementary Table 7. D' values extracted from the outlined regions are plotted in bar graphs. Green bars refer to statistical classification tests for each region. The significance of *t*-tests that compared *d'* values against chance level are also reported. *** $p < 0.001$; * $p < 0.05$. Error bars refer to bootstrap-based 95% confidence intervals.

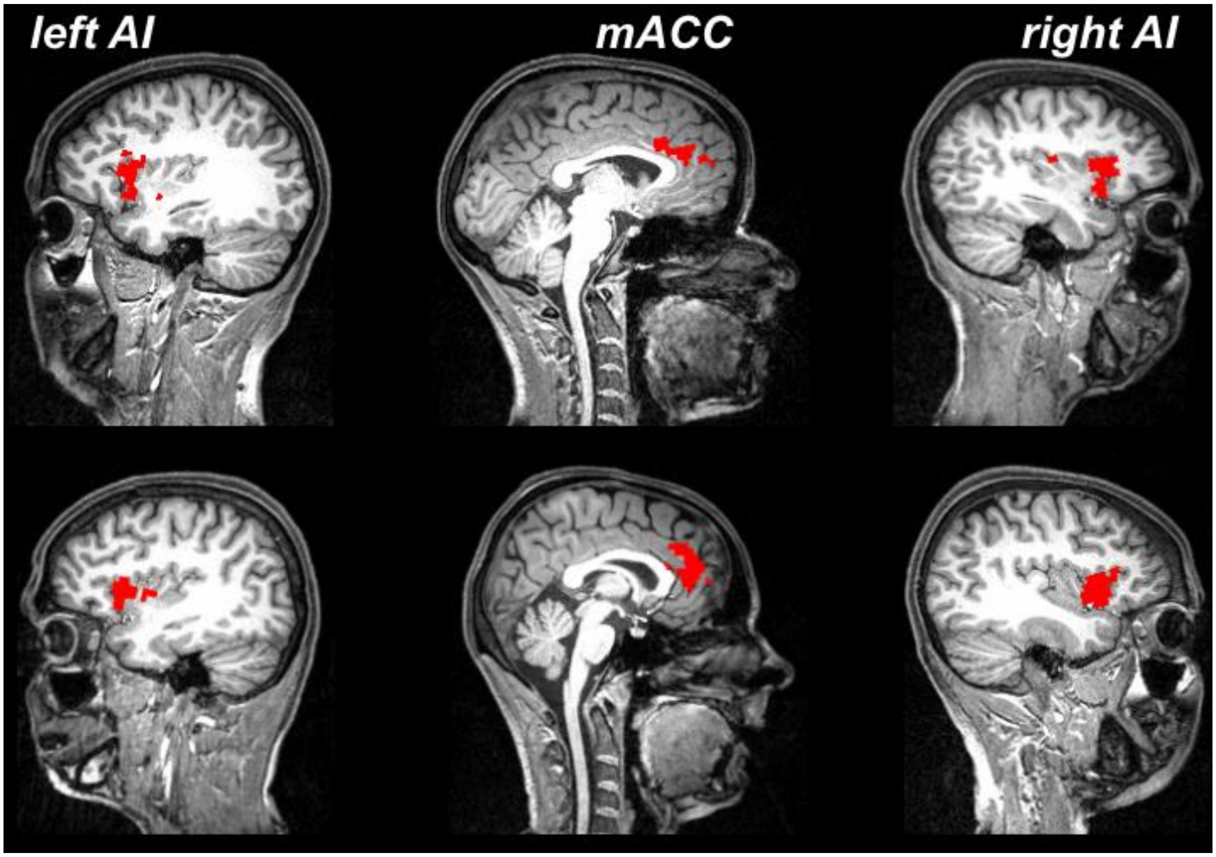


**Supplementary Figure 8. Graphical representation of the identified ROIs in the native brain of 6 representative subjects**

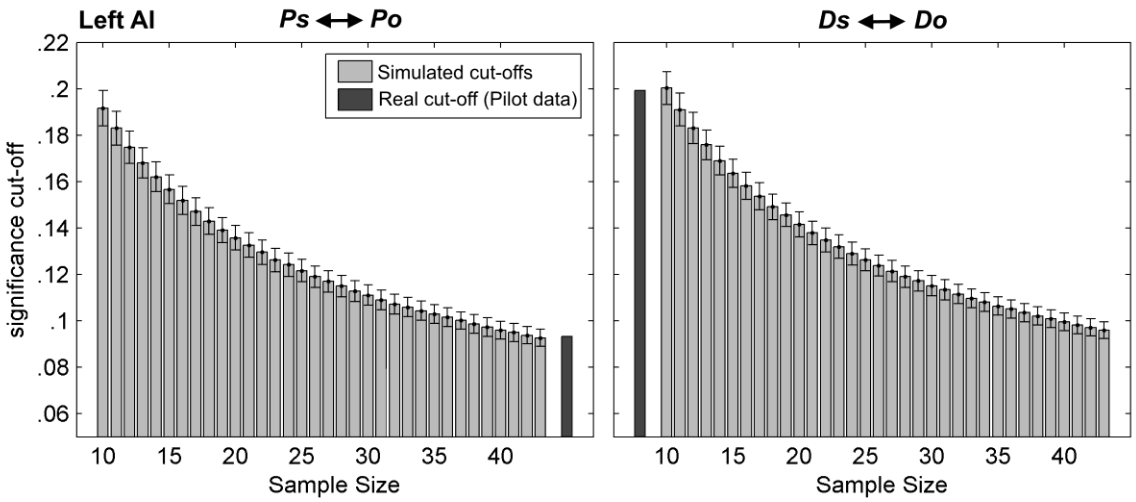


**Supplementary Figure 9. Monte Carlo simulation of significant cut-offs in the Pilot Data.** Graphical representation of the Monte Carlo simulations for two representative cross-target analyses for pain (Ps ↔ Po) and disgust (Ds ↔ Do) in left AI, which displays the simulated cut-offs (95 percentile in Permutation-based Null distribution of *d's*, illustrated in light grey) in a sample ranging from N=10 to N=43, together with the real cut-off from the pilot data illustrated in dark grey (N = 43 for Pain Pilot Data; N = 10 for Disgust Pilot Data). See Supplementary Note 2 for more details.

**Supplementary Table 1**. Within-task classification of first-person pain (Ps vs. nPs) and disgust (Ds vs. nDs). Coordinates (in standard MNI space) refer to maximally activated foci. L, Left hemisphere; R, right hemisphere; M, medial regions. *** p < 0.001, ** p < 0.01, * p < 0.05, permutation-based correction for multiple comparisons at the cluster level.

| | *SIDE* | *Coordinates* | | | $T_{(19)}$ | *Cluster size* |
|---|---|---|---|---|---|---|
| | | *x* | *Y* | *z* | | |
| ***Ps (vs. nPs)*** | | | | | | |
| Anterior Insula | R | 30 | 18 | -14 | 7.14 | |
| Posterior Insula | R | 38 | -18 | 12 | 6.53 | |
| Parietal Operculum | R | 50 | -22 | 22 | 7.24 | |
| Postcentral Gyrus | R | 56 | -22 | 36 | 10.99 | |
| Inferior Parietal Cortex | R | 54 | -56 | 42 | 7.05 | |
| Inferior Frontal Gyrus | R | 46 | 14 | 28 | 8.12 | |
| Middle Frontal Gyrus | R | 38 | 50 | 20 | 7.13 | |
| Amygdala | R | 22 | -4 | -12 | 6.02 | |
| Anterior Insula | L | -36 | 16 | -14 | 6.78 | |
| Posterior Insula | L | -48 | 4 | 4 | 6.21 | |
| Parietal Operculum | L | -54 | 4 | 8 | 7.36 | 120997*** |
| Postcentral Gyrus | L | -56 | -28 | 28 | 9.95 | |
| Inferior Parietal Cortex | L | -48 | -62 | 26 | 10.14 | |
| Inferior Frontal Gyrus | L | -50 | 14 | 14 | 7.47 | |
| Middle Frontal Gyrus | L | -30 | 54 | 22 | 7.55 | |
| Hippocampus/Amygdala | L | -18 | -18 | -18 | 5.63 | |
| Middle Cingulate Cortex | M | 0 | 20 | 34 | 6.96 | |
| Medial Prefrontal Cortex | M | -4 | 42 | 42 | 7.00 | |
| Posterior Cingulate Cortex | M | 2 | -50 | 28 | 6.27 | |
| Precuneus | M | 0 | -70 | 54 | 9.98 | |
| Lingual Gyrus | M | 4 | -78 | 0 | 10.90 | |
| ***Ds (vs. nDs)*** | | | | | | |
| Anterior Insula | R | 44 | 22 | -18 | 4.07 | |
| Middle Insula | R | 56 | 16 | -2 | 5.20 | |
| Precentral Gyrus | R | 54 | 4 | 44 | 6.19 | |
| Inferior Parietal Cortex | R | 54 | -60 | 40 | 7.32 | 55256*** |
| Middle Temporal Gyrus | R | 50 | -38 | 6 | 6.99 | |
| Inferior Frontal Gyrus | R | 46 | 40 | 22 | 4.98 | |
| Fusiform Gyrus | R | 24 | -62 | -20 | 7.42 | |
| Globus Pallidus | R | 14 | -6 | 6 | 4.83 | |

| | | | | | | |
|---|---|---|---|---|---|---|
| Precentral Gyrus | L | -58 | 2 | 20 | 7.00 | |
| Inferior Parietal Cortex | L | -60 | -38 | 42 | 7.51 | |
| Middle Temporal Gyrus | L | -50 | -58 | 8 | 7.29 | |
| Inferior Frontal Gyrus | L | -50 | 40 | 4 | 5.89 | |
| Fusiform Gyrus | L | -32 | -64 | -20 | 7.64 | |
| Middle Cingulate Cortex | M | -2 | 0 | 48 | 6.86 | 55256*** |
| Medial Prefrontal Cortex (*dorsal part*) | M | -14 | 48 | 38 | 5.57 | |
| Posterior Cingulate Cortex | M | -4 | -48 | 32 | 6.77 | |
| Precuneus | M | -6 | -56 | 52 | 5.68 | |
| Lingual Gyrus | M | -2 | -94 | -2 | 8.32 | |
| Globus Pallidus | L | -14 | -10 | 10 | 4.73 | 347* |

*** $p < 0.001$; ** $p < 0.01$; * $p < 0.05$ corrected for multiple comparisons

**Supplementary Table 2**. Cross-modal classification of first-person pain and disgust (Ps ↔ Ds). As cross-modal effects are meaningful only in regions with reliable signals for both first-hand pain and disgust, the analysis was focused only on those coordinates exhibiting significant effects for Ps (vs. nPs) and Ds (vs. nDs) in the within-task classification (conjunction with threshold of p < 0.05, uncorrected).

| | ***SIDE*** | ***Coordinates*** | | | $T_{(19)}$ | ***Cluster size*** |
|---|---|---|---|---|---|---|
| | | ***X*** | ***Y*** | ***z*** | | |
| ***Ps ↔ Ds*** (incl. masking *Ps ∩ Ds,* at $p < 0.05$ uncorr.) | | | | | | |
| Mid-Ant. Cing. Cortex (mACC) | M | -4 | 38 | 8 | 6.34 | 3106*** |
| Middle Cing. Cortex (MCC) | | 14 | 20 | 26 | 5.77 | |
| Anterior Insula (AI) | L | -30 | 28 | -10 | 6.21 | 1206*** |
| post. Sup. Temporal Sulcus (pSTS) | L | -54 | -62 | 24 | 5.36 | 400* |

*** $p < 0.001$; * $p < 0.05$ corrected for multiple comparisons

**Supplementary Table 3**. Within-task classification of painful events affecting others (Po vs. nPo) and cross-target classification testing for shared activity patterns between one's own and others' pain (Ps ↔ Po). As cross-target effects are meaningful only in regions with reliable signals for both first-hand and vicarious pain, the analysis was focused only on those coordinates exhibiting significant effects for Ps (vs. nPs) and Po (vs. nPo) in the within-task classification (conjunction with threshold of p < 0.05, uncorrected).

| | ***SIDE*** | ***Coordinates*** | | | $T_{(19)}$ | ***Cluster size*** |
|---|---|---|---|---|---|---|
| | | ***x*** | ***Y*** | ***z*** | | |
| ***Po (vs. nPo)*** | | | | | | |
| Superior Parietal Cortex | R | 20 | -66 | 46 | 7.41 | 37326*** |
| Inferior Parietal Cortex | R | 52 | -38 | 30 | 6.45 | |
| Middle Temporal Gyrus | R | 66 | -34 | -8 | 6.02 | |
| Superior Parietal Cortex | L | -22 | -58 | 52 | 10.86 | |
| Inferior Parietal Cortex | L | -56 | -34 | 20 | 8.02 | |
| Middle Temporal Gyrus | L | -64 | 44 | 0 | 5.03 | |
| Lingual Gyrus | M | 4 | -80 | -10 | 11.38 | |
| Precuneus | M | -4 | -62 | 46 | 8.74 | |
| Anterior Insula | R | 40 | 28 | -2 | 5.32 | 16064*** |
| Middle Frontal Gyrus | R | 40 | 50 | 22 | 4.39 | |
| Precentral Gyrus | L | -36 | -4 | 54 | 5.49 | |
| Anterior Insula | L | -44 | 24 | 2 | 4.96 | |
| Inferior Frontal Gyrus | L | -48 | 26 | 26 | 6.41 | |
| Middle Cingulate Cortex | M | -2 | 2 | 50 | 5.50 | |
| Medial Prefrontal Cortex | M | 6 | 36 | 36 | 8.90 | |
| Precentral Gyrus | R | 42 | 6 | 46 | 6.54 | 807** |
| Postcentral Gyrus | L | -54 | -6 | 20 | 5.81 | 276* |
| ***Ps ↔ Po*** (incl. masking *Ps ∩ Po*) | | | | | | |
| Middle Cingulate Cortex (MCC) | M | 14 | 22 | 34 | 5.97 | 857* |
| Superior Frontal Sulcus (SFS) | R | 26 | 46 | 38 | 5.39 | |
| Superior Frontal Sulcus | L | -20 | 40 | 20 | 4.66 | 304* |
| posterior Superior Temporal Sulcus (pSTS) | R | 44 | -44 | 8 | 5.15 | 312* |
| Postcentral Gyrus (PCG) | L | -62 | -16 | 28 | 6.33 | 172 |
| Anterior Insula (AI) | L | -44 | 20 | 8 | 4.48 | 131 |

*** $p < 0.001$; ** $p < 0.01$; * $p < 0.05$ corrected for multiple comparisons

**Supplementary Table 4**. Within-task classification of disgusting events affecting others (Do vs. nDo) and cross-target classification testing for shared activity patterns between one's own and others' disgust (Ds ↔ Do). As cross-target effects are meaningful only in regions with reliable signals for both first-hand and vicarious disgust, the analysis was focused only on those coordinates exhibiting significant effects for Ds (vs. nDs) and Do (vs. nDo) in the within-task classification (conjunction with threshold of $p < 0.05$, uncorrected).

| | ***SIDE*** | ***Coordinates*** | | | $T_{(19)}$ | ***Cluster size*** |
|---|---|---|---|---|---|---|
| | | ***x*** | ***y*** | ***z*** | | |
| ***Do (vs. nDo)*** | | | | | | |
| Inferior Occipital Gyrus | R | 32 | -82 | -10 | 10.92 | $19749^{***}$ |
| Inferior Occipital Gyrus | L | -28 | -84 | -14 | 6.22 | |
| Angular Gyrus | L | -40 | -64 | 40 | 4.99 | |
| Lingual Gyrus | M | 8 | -78 | 2 | 9.29 | |
| Retrosplenial Cortex | M | 4 | -58 | 14 | 5.83 | |
| Precuneus | M | 4 | -66 | 58 | 5.67 | |
| Dorsolateral Prefrontal Cortex | R | 40 | 52 | 12 | 5.51 | $8810^{***}$ |
| Inferior Frontal Gyrus | L | -44 | 24 | -8 | 4.78 | |
| Dorsolateral Prefrontal Cortex | L | -30 | 56 | 8 | 5.83 | |
| Medial Prefrontal Cortex | M | -12 | 62 | 16 | 5.86 | |
| Superior Temporal Sulcus | R | 60 | -34 | 0 | 5.49 | $3901^{***}$ |
| Supramarginal Gyrus | R | 58 | -22 | 26 | 4.55 | |
| Postcentral Gyrus | R | 56 | 4 | 22 | 5.71 | |
| Anterior Insula (AI) | R | 54 | 16 | -6 | 6.43 | |
| Inferior Frontal Gyrus | R | 40 | 38 | -12 | 4.19 | |
| Middle Temporal Gyrus | L | -62 | -14 | -18 | 5.17 | $450^{*}$ |
| Superior Temporal Sulcus | L | -62 | -8 | 4 | 4.52 | |
| ***Ds ↔ Do*** (incl. masking *Ds* ∩ *Do*) | | | | | | |
| Inferior Occipital Gyrus | R | 26 | -94 | -6 | 5.10 | $1286^{***}$ |
| Calcarine cortex | M | -2 | -92 | 6 | 5.42 | |
| Posterior Cingulate Cortex | M | 2 | -50 | 22 | 7.23 | $649^{**}$ |

*** $p < 0.001$; ** $p < 0.01$; * $p < 0.05$ corrected for multiple comparisons

**Supplementary Table 5**. Brain regions associated with reliable cross-modal responses across targets for pain and disgust (Ps ↔ Do and Ds ↔ Po). This measure was achieved by combining together Ps ↔ Do and Ds ↔ Po. Because cross-modal cross-target effects in absence of significant cross-target and cross-modal classifications would hardly be interpretable, our analysis was restricted to regions that were identified for the conjoint effect of Ps ↔ Ds ∩ Ps ↔ Po ∩ Ds ↔ Do (at p< 0.05 uncorrected).

| | *SIDE* | *Coordinates* | | | $T_{(19)}$ | *Cluster size* |
|---|---|---|---|---|---|---|
| | | *x* | *y* | *z* | | |
| ***mean(Ps ↔ Do, Ds ↔ Po)*** (incl. masking *Ps ↔ Ds ∩ Ps ↔ Po ∩ Ds ↔ Do,* at $p < 0.05$ uncorr.) | | | | | | |
| Inferior Frontal Gyrus (IFG) | R | 50 | 30 | 10 | 5.29 | 154** |
| Inferior Frontal Gyrus (IFG) | L | -38 | -34 | 6 | 4.52 | 87* |
| Lateral Orbital Gyrus | R | 32 | 26 | -12 | 4.51 | 112* |
| Middle Cingulate (Cortex MCC) | M | -2 | 20 | 40 | 4.90 | 112* |

** $p < 0.01$; * $p < 0.05$ corrected for multiple comparisons

**Supplementary Table 6**. Within-task classification of unfair events affecting the self (Us vs. Ms) and the other (Uo vs. Mo)

| | *SIDE* | *Coordinates* | | | $T_{(19)}$ | *Cluster size* |
|---|---|---|---|---|---|---|
| | | *x* | *y* | *z* | | |
| ***Us (vs. Ms)*** | | | | | | |
| Ventromedial Pref. Cortex | M | -4 | 48 | -4 | 7.41 | |
| Dorsomedial Pref. Cortex | M | -6 | 54 | 16 | 7.81 | |
| Middle Cingulate Cortex | M | 6 | 2 | 46 | 8.16 | |
| Post. Cing. Cortex/Precuneus | M | 6 | -66 | 34 | 8.66 | |
| Calcarine Sulcus | M | -4 | -86 | -8 | 6.88 | 38251*** |
| Parieto-Occipital Cortex | R | 42 | -78 | 34 | 6.19 | |
| Inferior Parietal Cortex | R | 62 | -52 | 36 | 6.92 | |
| Parieto-Occipital Cortex | L | -30 | -78 | 34 | 11.85 | |
| Inferior Parietal Cortex | L | -64 | -34 | 30 | 5.71 | |
| Ant. Insula/Inf. Front. Gyrus | R | 50 | 26 | -8 | 4.27 | 309* |
| Post. Insula | L | -45 | 0 | -8 | 5.27 | |
| Ant. Insula (*ventral part*) | L | -40 | 18 | -26 | 7.01 | 1178** |
| Ant. Insula/Inf. Front. Gyrus | L | -42 | 22 | -2 | 3.95 | |
| Precentral Gyrus | R | 36 | 4 | 52 | 6.52 | 920** |
| Middle Frontal Gyrus | R | 44 | 44 | 12 | 6.05 | 762* |
| Precentral Gyrus | L | -46 | -4 | 54 | 4.97 | 1324** |
| Middle Frontal gyrus | L | -32 | 34 | 40 | 5.44 | |
| Lingual Gyrus | R | 26 | -26 | -26 | 5.92 | 745* |
| Fusiform Gyrus | R | 24 | -46 | -2 | 4.72 | |
| Inferior Temporal Sulcus | L | -52 | -16 | -20 | 5.39 | 483* |
| Superior Temporal Sulcus | L | -54 | -18 | -2 | 5.37 | |
| ***Uo (vs. Mo)*** | | | | | | |
| Ventromedial Pref. Cortex | M | -2 | 54 | -6 | 6.40 | |
| Dorsomedial Pref. Cortex | M | 0 | 54 | 36 | 5.98 | |
| Middle Cingulate Cortex | M | -2 | 6 | 32 | 4.99 | |
| Ventral Striatum | M | 4 | 10 | 0 | 4.97 | 13747*** |
| Middle Frontal Gyrus | R | 32 | 54 | 26 | 7.43 | |
| Middle Frontal Gyrus | L | -36 | 36 | 40 | 6.30 | |
| Ant. Insula/Inf. Front. Gyrus | L | -28 | 32 | 12 | 4.69 | |
| Ant. Insula/Inf. Front. Gyrus | R | 54 | 16 | -8 | 5.56 | 309* |
| Post. Cing. Cortex/Precuneus | M | 6 | -44 | 44 | 6.90 | |
| Lingual Gyrus | M | -2 | -72 | -2 | 8.96 | |
| Inferior Parietal Cortex | R | 56 | -50 | 40 | 5.36 | 23428*** |
| Inferior Parietal Cortex | L | -42 | -44 | 52 | 5.31 | |
| Middle Temporal Gyrus | L | -56 | -40 | -8 | 7.13 | |

| | | | | | | |
|---|---|---|---|---|---|---|
| Cerebellum | R | 36 | -70 | -28 | 5.23 | 541* |
| Postcentral Gyrus | L | -60 | -18 | 38 | 4.37 | 241* |

*** $p < 0.001$; ** $p < 0.01$; * $p < 0.05$ corrected for multiple comparisons

**Supplementary Table 7**. Cross-target classification testing for shared activity patterns for self-related and other-related unfairness (Us ↔ Uo), and cross-modal classification testing for shared activity patterns for self-disgust and others' unfairness (Ds ↔ Uo). As for the previous cross-target and cross-modal classifications, we focused our analysis on those coordinates exhibiting significant effects in the within-task classifications as identified in a conjunction analysis thresholded at $p < 0.05$ (uncorrected).

| | ***SIDE*** | ***Coordinates*** | | | $T_{(19)}$ | ***Cluster size*** |
|---|---|---|---|---|---|---|
| | | ***x*** | ***y*** | ***z*** | | |
| ***Us ↔ Uo*** (incl. masking *Us ∩ Uo*) | | | | | | |
| Anterior Cingulate Cortex (ACC) | M | -4 | 36 | -14 | 4.48 | 5324*** |
| Dorsomedial Pref. Cortex (dMPFC) | M | -8 | -38 | 38 | 6.73 | |
| Middle Frontal gyrus | R | 24 | 30 | 48 | 5.04 | |
| Middle Frontal gyrus | L | -30 | 34 | 40 | 4.94 | |
| Anterior Insula (AI) | R | 42 | 14 | 6 | 4.76 | 253* |
| Ant. Ins./Inf. Front. Gyrus (AI/IFG) | L | 38 | 26 | 8 | 4.06 | 414* |
| Inferior Frontal Gyrus | L | -44 | 12 | 20 | 4.42 | |
| Inferior Parietal Cortex (IPC) | R | 46 | -56 | 30 | 4.42 | 397* |
| Inferior Temporal Gyrus | R | 50 | -52 | -18 | 6.00 | 656** |
| Inferior Parietal Cortex | L | -44 | -46 | 38 | 9.52 | 3744*** |
| Intraparietal Sulcus (*caud. part*) | L | -34 | -70 | 34 | 6.54 | |
| Sup. Temp. Sulcus (*post. part*) | L | -44 | -48 | 6 | 5.81 | |
| Post. Cing. Cortex/Precuneus (PCC) | M | -10 | -56 | 44 | 5.91 | 4483*** |
| Calcarine Gyrus | M | -2 | -100 | 6 | 9.00 | |
| ***Ds ↔ Uo*** (incl. masking *Ds ∩ Uo*) | | | | | | |
| Anterior cingulate Cortex/Medial Pref. Cortex (ACC) | M | -8 | 50 | 8 | 6.21 | 1254*** |
| Ant. Ins./Inf. Front. Gyrus (AI/IFG) | L | -36 | 32 | 10 | 4.83 | |

*** $p < 0.001$; ** $p < 0.01$; * $p < 0.05$ corrected for multiple comparisons

**Supplementary Table 8**. Comparison between results from Pilot data (described in Supplementary Note 1) and the Main data (described in the main text). D' values, and significance cut-offs are reported, with bold values corresponding to effects associated with p < 0.05 uncorrected. For pilot data, the expected significant cut-off for a population of N = 19 is also reported (see Supplementary Note 2).

| | Pilot data | | Main data |
|---|---|---|---|
| | *d' [cut-off]* | *Expected cut-off at N = 19* | *d' [cut-off]* |
| **Left AI** | | | |
| *Ps (vs. nPs)* | **1.20** [0.24]* | **[0.27]*** | **0.98** [0.24]* |
| *Po (vs. nPo)* | **0.48** [0.10]* | **[0.14]*** | **0.49** [0.22]* |
| *Ps ↔ Po* | **0.17** [0.09]* | **[0.14]*** | **0.21** [0.16]* |
| *Ds (vs. nDs)* | **0.35** [0.30]* | **[0.21]*** | **0.61** [0.22]* |
| *Do (vs. nPo)* | 0.26 [0.29]† | **[0.20]*** | **0.24** [0.19]* |
| *Ds ↔ Do* | **0.20** [0.19]* | **[0.15]*** | **0.19** [0.15]* |
| **Right AI** | | | |
| *Ps (vs. nPs)* | **1.40** [0.23]* | **[0.27]*** | **1.29** [0.25]* |
| *Po (vs. nPo)* | **0.56** [0.09]* | **[0.13]*** | **0.36** [0.22]* |
| *Ps ↔ Po* | **0.13** [0.09] * | [0.14] | 0.11 [0.17] |
| *Ds (vs. nDs)* | 0.29[0.31]† | **[0.20]*** | **0.49** [0.26]* |
| *Do (vs. nPo)* | **0.28** [0.27]* | **[0.20]*** | **0.40** [0.21]* |
| *Ds ↔ Do* | 0.02 [0.19] | [0.15] | 0.12 [0.14] |
| **mACC** | | | |
| *Ps (vs. nPs)* | **0.79** [0.24]* | **[0.27]*** | **1.61** [0.25]* |
| *Po (vs. nPo)* | **0.44** [0.09]* | **[0.14]*** | **0.46** [0.20]* |
| *Ps ↔ Po* | **0.15** [0.10]* | **[0.14]*** | **0.35** [0.17]* |
| *Ds (vs. nDs)* | **0.60** [0.29]* | **[0.22]*** | **0.65** [0.22]* |
| *Do (vs. nPo)* | **0.36** [0.28]* | **[0.20]*** | **0.53** [0.20]* |
| *Ds ↔ Do* | **0.23** [0.20]* | **[0.15]*** | **0.25** [0.15]* |

* $p < 0.05$, † $p < 0.07$

**Supplementary Note 1**

The sensitivity of the Pain task and Disgust task was assessed through an analysis of two independent Pilot datasets. In particular, the Disgust task was piloted through an independent dataset of N=10 (from a separate project). Stimulus selection, stimuli and experimental set-up closely matched those implemented in the main Disgust task, with 3 exceptions: (*i*) participants were asked to taste gustatory stimuli for 6 s (compared to 4s) before swallowing, (*ii*) only 50% of stimuli were followed by ratings of experienced unpleasantness, (*iii*) an anonymous partner acted as confederate.

The robustness of effects associated with the Pain task was assessed from an independent dataset of N=43, based on the same experiment described by Corradi-Dell'Acqua et al.[1]. In this task, individuals underwent two independent experimental sessions: a first-hand pain session, where painful temperatures (and painless controls) were delivered on the palms; a vicarious pain condition, in which individuals saw photographs of hands in pain (or control hands in painless contexts). Stimuli selection, and experimental set-up matched closely those implemented in our previous study[1], with one exception: whereas in our previous study first-hand pain was delivered only on the right[1], in the present dataset pain was delivered to both sides.

Functional images associated with these pilot data (recorded with Siemens Trio/Verio 3T scanner) were fed to the same processing pipelines and ROI-based classification procedures described in the method section of the manuscript. Supplementary Table 8 depicts the *d'* values from the pilot data in comparison with those of the corresponding tests from the main experiment. Overall pilot data led to results highly compatible with those found in the main experiment, with higher-than-chance within-task classifications in all 3 ROIs and cross-target classifications in left AI and mACC (note that some effects associated with the independent Disgust task were only marginally significant, though probably due to the sample size of only N=10, but they clearly conformed to the predicted effects). This speaks in favor of the robustness of our results, as they generalize to independent data and (in the case of the Pain task) even to different kinds of painful stimuli. The only difference between the pilot and the main experiments relates to the cross-target classification *Ps* ↔ *Po* in right AI, which led to a small, but significant, *d'* in the pilot pain data.

**Supplementary Note 2**

To explicitly address whether the group-wise *d'*s observed on the Pilot data would have been significant under N=19, a Monte Carlo simulation was run to estimate plausible significance cut-offs under different population sizes. For each classification analysis, 1000 simulations were conducted in order to obtain arrays of *d'*s of the desired sample size and with the same distributional characteristics of the permutation-based *d'*s from the pilot dataset. These simulated data were used to calculate 1000 group-wise significance ($95^{th}$ percentile) cut-offs, the average of which is reported in Supplementary Table 8. An exemplary graphical representation of the Monte Carlo simulation for two representative cross-target analyses is provided in Supplementary Figure 9, which displays the simulated cut-offs (95 percentile in Permutation-based Null distribution of d's, illustrated in light grey) in a sample ranging from N=10 to N=43, together with the real cut-off from the pilot data illustrated in dark grey (N=43 for Pain; N=10 for Disgust). The close correspondence of simulated and real cut-offs indicates that simulations provide good estimates of critical statistical parameters achieved for various sample sizes.

As clearly visible from the results in Supplementary Table 8, although some of the *d'*s from the Pilot data were not or only marginally (disgust pilot data) significant, the Monte Carlo simulation confirms that a positive effect could nonetheless reliably be observed with a population of N=19. The only exception is provided by the *d'* associated with cross-target classification *Ds* ↔ *Do* for right AI (d' = 0.02): for this specific case it is unlikely that the absence reliable classification could be related to the sample size, as no significance would be expected even when simulating a population of N=100.

## Supplementary References

1. Corradi-Dell'Acqua, C., Hofstetter, C. & Vuilleumier, P. Felt and Seen Pain Evoke the Same Local Patterns of Cortical Activity in Insular and Cingulate Cortex. *J. Neurosci.* **31,** 17996 – 18006 (2011).
